# Fine-mapping the immunodominant antibody epitopes on consensus sequence-based HIV-1 envelope trimer vaccine candidates

**DOI:** 10.1038/s41541-022-00576-9

**Published:** 2022-11-25

**Authors:** E. I. M. M. Reiss, M. M. van Haaren, J. van Schooten, M. A. F. Claireaux, P. Maisonnasse, A. Antanasijevic, J. D. Allen, I. Bontjer, J. L. Torres, W-H Lee, G. Ozorowski, N. Vázquez Bernat, M. Kaduk, Y. Aldon, J. A. Burger, H. Chawla, A. Aartse, M. Tolazzi, H. Gao, P. Mundsperger, M. Crispin, D. C. Montefiori, G. B. Karlsson Hedestam, G. Scarlatti, A. B. Ward, R. Le Grand, R. Shattock, N. Dereuddre-Bosquet, R. W. Sanders, M. J. van Gils

**Affiliations:** 1 Amsterdam UMC, location University of Amsterdam, Department of Medical Microbiology and Infection Prevention, Laboratory of Experimental Virology, Meibergdreef 9, Amsterdam, The Netherlands; 2 Université Paris-Saclay – CEA - INSERM U1184, Center for Immunology of Viral, Auto-immune, Hematological and Bacterial diseases (IMVA-HB/IDMIT), Fontenay-aux-Roses, France; 3 Department of Integrative Structural and Computational Biology, Scripps Consortium for HIV/AIDS Vaccine Development (CHAVD), The Scripps Research Institute, La Jolla, CA USA; 4 School of Biological Sciences, University of Southampton, Southampton, UK; 5 Department of Microbiology, Tumor and Cell Biology, Karolinska Institutet, Stockholm, Sweden; 6 Department of Virology, Biomedical Primate Research Centre, Rijswijk, the Netherlands; 7 Viral Evolution and Transmission Unit, Division of Immunology, Transplantation and Infectious Diseases, IRCCS Ospedale San Raffaele, Milan, Italy; 8 Department of Surgery, Duke University Medical Center, Durham, NC USA; 9 Polymun Scientific Immunbiologische Forschung GmbH, Klosterneuburg, Austria; 10 Division of Mucosal Infection and Immunity, Department of Medicine, Imperial College of Science, Technology and Medicine, London, UK; 11 Department of Microbiology and Immunology, Weill Medical College of Cornell University, New York, NY USA

**Keywords:** Protein vaccines, Virology

## Abstract

The HIV-1 envelope glycoprotein (Env) trimer is the key target for vaccines aimed at inducing neutralizing antibodies (NAbs) against HIV-1. The clinical candidate immunogen ConM SOSIP.v7 is a stabilized native-like HIV-1 Env trimer based on an artificial consensus sequence of all HIV-1 isolates in group M. In preclinical studies ConM SOSIP.v7 trimers induced strong autologous NAb responses in non-human primates (NHPs). To fine-map these responses, we isolated monoclonal antibodies (mAbs) from six cynomolgus macaques that were immunized three times with ConM SOSIP.v7 protein and boosted twice with the closely related ConSOSL.UFO.664 immunogen. A total of 40 ConM and/or ConS-specific mAbs were isolated, of which 18 were retrieved after the three ConM SOSIP.v7 immunizations and 22 after the two immunizations with ConSOSL.UFO.664. 22 mAbs (55%) neutralized the ConM and/or ConS virus. Cross-neutralization of ConS virus by approximately one-third of the mAbs was seen prior to ConSOSL.UFO.664 immunization, albeit with modest potency. Neutralizing antibodies predominantly targeted the V1 and V2 regions of the immunogens, with an apparent extension towards the V3 region. Thus, the V1V2V3 region is immunodominant in the potent NAb response elicited by two consensus sequence native-like HIV-1 Env immunogens. Immunization with these soluble consensus Env proteins also elicited non-neutralizing mAbs targeting the trimer base. These results inform the use and improvement of consensus-based trimer immunogens in combinatorial vaccine strategies.

## Introduction

Four decades into the HIV/AIDS pandemic, 40.1 million people have died from AIDS-related illnesses^1^. Even with the present-day availability of effective measures such as antiretroviral treatment (ART), undetectable = untransmittable (U = U) practices, and pre-exposure prophylaxis (PrEP), human immunodeficiency virus 1 (HIV-1) infects 1.5 million persons annually, stressing the global need for a protective HIV-1 vaccine.

A major challenge in the vaccine quest is developing an effective immunogen or combination of immunogens capable of generating broad protection against the extensive variety of circulating HIV-1 variants. These variants can be divided into four different groups (M, N, O, and P), with viruses in HIV-1 group M being the driving force behind the current pandemic. Viruses in this group can be subdivided into nine subtypes (A–K)^2,3^ which each have specific geographical localizations. For instance, subtype B can be predominantly found in Europe and the Americas, whereas subtype C is dominant in Southern Africa and Asia^3^. In addition, recombination between these subtypes adds immensely to the diversity of circulating strains^3^. These circulating and unique recombinant forms (CRFs and URFs, respectively) also tend to follow a geographical distribution, e.g., with CRF01_AE as a dominant variant in Southeast Asia and CRF02_AG in West Africa^4^. Most of the observed variability between subtypes is due to differences in the envelope glycoprotein complex (Env). Env diversity between subtypes of HIV-1 can be as much as 35%^5^. Antibodies (Abs) that target conserved determinants of the virus could help to overcome this challenge. Such broadly neutralizing antibodies (bNAbs) develop during natural HIV-1 infection in a subset of patients and have been shown to protect non-human primates (NHPs) from infection with simian-human chimeric viruses (SHIVs) when administered at the mucosal site of virus entry^6^, as well as in passive immunization studies. In these studies, modest serum neutralization titers brought about by potent bNAbs were sufficient to prevent virus acquisition^7^. More recently, the Antibody Mediated Protection (AMP) trials (HIV Vaccine Trials Network (HVTN) 704/HIV Prevention Trials Network (HPTN) 085 and HVTN 703/HPTN 081), two large passive immunization studies in humans using the single bNAb VRC01, showed a 75.4% prevention efficacy from VRC01 sensitive viruses^8^, although the overall efficacy was low because of abundant circulation of VRC01-resistant viruses.

Several epitopes on Env are targeted by bNAbs, including the fusion peptide, the membrane proximal external region (MPER), the CD4 binding site (CD4bs), and the gp120-gp41 interface region^9^. Variable regions can also be targeted by bNAbs if they include conserved subregions and/or conserved glycans^10,11^. Current HIV-1 immunogens, in particular those based on native-like Env trimers with SOSIP stabilizing mutations, elicit autologous neutralizing Abs (NAbs) in animal models. In contrast, the induction of neutralization breadth is rare^12–14^ and only reported after elaborate immunization schemes^15–17^. Characterization of the Ab responses to these immunogens demonstrated that each immunogen possesses strain-specific, immunodominant antigenic determinants^18,19^. Strain-specific holes in the glycan shield surrounding the trimer, and the Env trimer base are especially immunogenic. These strain-specific Ab responses seem to dominate the response to the immunogen, possibly hindering the development of bNAbs.

A potential way to overcome subtype variability in an immunogen is the use of artificial sequences, the rationale being to limit strain-specific antigenic determinants that may hinder responses to bNAb epitopes. There are two strategies to design proteins with conserved epitopes, either mosaic or consensus proteins. Mosaic proteins combine optimal epitopes for bNAbs in the sequence, whereas consensus proteins take the most prevalent amino acid in each position. In the past, two consensus sequences of HIV-1 group M viruses were created using different methods. One consensus sequence, named Consensus S (ConS), is based on all the known sequences in group M^20,21^. The other, Consensus M (ConM), is based on the consensus sequences generated for each subtype in group M^22^. Both ConS and ConM lack strain-specific glycan holes, contain few rare amino acids, and have relatively short variable loop regions. A priori, these characteristics appear to be favorable to prevent the development of dominant autologous NAbs^23^, and consensus immunogens might therefore be attractive components in a vaccination regimen.

To assess the immunological potential of consensus Env trimers in vivo, preclinical studies in rabbits and NHPs were previously performed. The stabilized cleaved pre-fusion ConM SOSIP.v7 trimer was capable of eliciting strong NAb responses against the autologous virus in rabbits and macaques and a rabbit immunization study demonstrated that the uncleaved pre-fusion optimized (UFO) ConSOSL.UFO.664 trimer successfully induced autologous neutralization^20,22^, although it should be noted that the ConM- and ConS-sequence-derived viruses are quite sensitive to neutralization (Tier 1A and Tier 1B, respectively). Based on these findings the consensus Env trimers ConM SOSIP.v7 and ConSOSL.UFO.664 have proceeded into the clinical testing stage. Early phase clinical trials in the United Kingdom and the Netherlands (ClinicalTrials.gov Identifiers: NCT03816137, NCT03961438, and NCT04046978) are currently underway, of which the first two (partially) overlap with the work described here in terms of immunogens, immunization schemes and adjuvants used. Gaining detailed information on the precise nature of B cell responses against these immunogens, including their immunodominant epitope(s) is valuable for predicting and evaluating the outcome of clinical studies and for accelerating iterative improvement of consensus Env immunogens.

In the study described here, we isolated monoclonal antibodies (mAbs) from NHPs after three priming immunizations with the soluble ConM SOSIP.v7 Env immunogen^22^, and again after two subsequent boosts with ConSOSL.UFO.664 Env immunogen^20^. MAbs isolated from subsequent time points provided the ability to follow (N)Ab development over time. We observed that NAbs predominantly targeted the variable 1 (V1) and variable 2 (V2) regions of the ConM SOSIP.v7 and ConSOSL.UFO.664 immunogens. We also report that, similar to other immunization studies using soluble Env trimers^24–26^, many non-NAbs targeted the base of the Env trimer. The findings reported here may be used to improve the performance of consensus immunogens.

## Results

### Consensus sequences-based Env trimers induced autologous NAb responses in NHPs

**Fig. 1:**
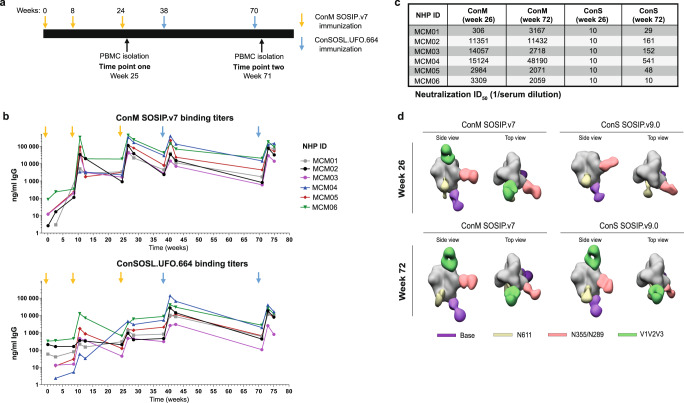
ConM SOSIP.v7 and ConSOSL.UFO.664 immunogens induce strong serological responses in NHPs. **a** Immunization and PBMC isolation schedule. **b** ConM SOSIP.v7-specific and ConSOSL.UFO.664-specific serum IgG binding titers (ng/ml) developed following ConM SOSIP.v7 (yellow arrows) and ConSOSL.UFO.664 protein immunizations (blue arrows). **c** ConM and ConS serum neutralization titers at week 26 and week 72 for six immunized NHPs. Neutralization ID_50_ is shown for each animal (1/serum dilution). **d** Antibody specificities were determined by EM-based polyclonal epitope mapping (EMPEM). Purified Fabs from week 26 and week 72 sera were complexed with either ConM SOSIP.v9.0 or ConS SOSIP.v9.0 trimers. Composite figures of the various antibody specificities detected by the 3D analysis are shown for all NHPs combined. Antibodies were detected targeting the V1V2V3 interface, the N611-glycan region, the N355/N289-glycan epitopes, and the base of the trimers.

Our goal was to characterize the antibody responses elicited by group M consensus trimer immunizations. To this end, we isolated mAbs from six cynomolgus macaques that were immunized three times with ConM SOSIP.v7 protein (Fig. 1a). After the three initial ConM SOSIP.v7 immunizations, the animals received two additional immunizations with ConSOSL.UFO.664 trimers. Strong ConM SOSIP.v7-specific antibody binding titers developed in all animals following immunization. Modest ConSOSL.UFO.664-specific antibody binding titers developed following the ConM SOSIP.v7 protein immunizations and were enhanced by each ConSOSL.UFO.664 protein boost (Fig. 1b). After three immunizations all six NHPs developed autologous ConM virus neutralization, five with very high titers (Fig. 1c). Following the two ConSOSL.UFO.664 protein boosts, the ID_50_ values remained consistently above 2000 for all animals. In contrast, ConS virus neutralization was absent prior to the ConSOSL.UFO.664 immunization, but developed in five out of six animals although at low to moderate titers (ID_50_ values between 29 and 541; Fig. 1c).

We next determined the dominant specificities of antibodies elicited by ConM SOSIP.v7 and ConSOSL.UFO.664 immunogens by using the EM-based polyclonal epitope mapping (EMPEM) technique, whereby total serum IgG is digested into antigen binding fragments (Fab), complexed with Env SOSIP trimers and imaged using negative-stain electron microscopy (NS-EM)^27^. Fab samples were prepared from week 26 sera and week 72 sera (i.e., two weeks after three ConM SOSIP.v7 and two ConSOSL.UFO.664 protein immunizations, respectively). Purified Fabs were complexed with either ConM SOSIP.v9.0 or ConS SOSIP.v9.0 trimers which contain additional stabilizing mutations that prevent trimer dissociation upon Fab binding (Supplementary Fig. 1a)^28^. Composite figures of the various antibody specificities detected by the 3D analysis are shown for each animal (Supplementary Fig. 2a), as well as relative frequencies per time point and Env protein (Supplementary Fig. 2b). Due to limited availability of sera for EMPEM analysis we were restricted to use a smaller amount than usual of purified Fab to complex with the ConM SOSIP.v9.0 and ConS SOSIP.v9.0 trimers. Therefore, we cannot exclude that some antibody specificities may have gone undetected by our 3D analysis, and we can only draw conclusions about the most dominant specificities. It might also explain some inconsistencies in the detection of responses against the N611 region that were detected with one protein and not the other, although we cannot exclude potential underlying N611 glycan occupancy differences on the ConM and ConS reagents (Supplementary Fig. 2a). For simplicity, we show composite figures of the various antibody specificities elicited in all the animals combined, where one Fab representative for each antibody specificity is presented if it was detected in at least one of the animals (Fig. 1d).

After three immunizations with ConM SOSIP.v7 protein, antibodies were detected targeting the V1V2V3 domain, a region surrounding the N355/N289 glycan epitopes, the base of the ConM SOSIP.v9.0 trimer and a region surrounding the N611 glycan. Abs binding to the latter likely target a hole in the glycan shield created by the under-occupancy of the N611 glycosylation sequon^29^. Overall, the same specificities, with the exception of the V1V2V3 interface, were also observed when week 26 Fabs were complexed with ConS SOSIP.v9.0 protein. After the additional two ConSOSL.UFO.664 protein boosts, antibodies targeting the V1V2V3 interface on the ConS SOSIP.v9.0 immunogen were also detected. We did not observe any additional antibody specificities after the two final immunizations with ConSOSL.UFO.664 protein at week 72 compared to week 26 at this level of resolution.

### Monoclonal antibodies reactive with ConM SOSIP.v7 and ConSOSL.UFO.664 were isolated from six NHPs

**Fig. 2:**
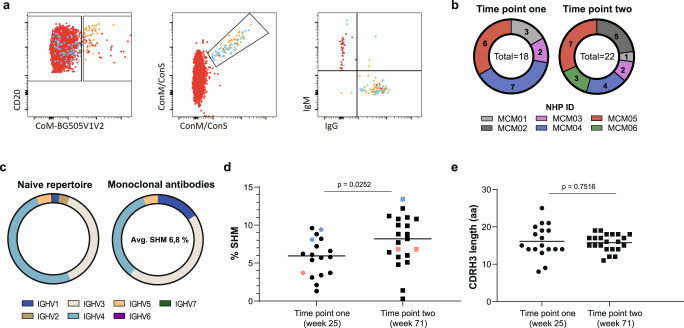
Characteristics of mAbs isolated from ConM SOSIP.v7 and ConSOSL.UFO.664 immunized NHPs. **a** For the immunized NHPs, Env-specific B cells were selected by fluorescence activated cell sorting using three differently labeled SOSIP trimers. ConM-BG505V1V2 protein was used in order to enrich for the V1V2 focused response. The same two fluorescent labels were conjugated to ConM SOSIP.v7 and ConSOSL.UFO.664 protein to reduce nonspecific background binding, while ConM SOSIP.v7- and/or ConSOSL.UFO.664-reactive cells could be identified. The gating strategy of one of the NHPs is shown here. **b** Absolute number of mAbs isolated per animal at time point one (week 25) and time point two (week 71). **c** The naive HC V-gene repertoire of each individual NHP was mapped through IgDiscover and categorized per IGHV family (IGHV1–IGHV7) (left). HC V-gene usage for the isolated mAbs was similarly mapped and the average level of SHM for all mAbs combined calculated (right). **d** The average level of SHM in the HC for mAbs isolated at the first time point (5.8% (range: 1.3–9.6%)) increases significantly at the second time point (8.2% (range: 0.3–13.4%)) (*p* = 0.0252). Differences were determined by two-tailed unpaired t-test. Two Ab lineages displaying V-gene similarities are depicted in blue (lineage CM05A) and pink (lineage CM04A). **e** Mean CDRH3 length (in amino acids) for mAbs isolated from all NHPs combined, compared between time point one and two. Differences were determined by two-tailed unpaired t-test (*p* = 0.7516).

To study ConM SOSIP.v7 protein and/or ConSOSL.UFO.664 protein targeting Abs at the monoclonal level, we sorted single antigen-specific B cells of the six NHPs at week 25 (time point one), i.e., after the third ConM SOSIP.v7 immunization, and week 71 (time point two), i.e., after the second ConSOSL.UFO.664 immunization, from peripheral blood mononuclear cells (PBMCs) (Fig. 1a). The probes used for sorting included the two immunogens ConM SOSIP.v7 and ConSOSL.UFO.664, as well as the chimeric ConM-BG505V1V2 SOSIP.v7 protein, essentially consisting of ConM SOSIP.v7 displaying the BG505 V1V2 region^22^ (Fig. 2a). The chimeric ConM-BG505V1V2 protein was used in order to enrich for V1V2-directed specificities through negative selection since such specificities were previously determined to be dominant in polyclonal NAb responses after ConM SOSIP.v7 immunization in rabbits^22,30^. To allow optimal identification of cells reactive with the ConM SOSIP.v7 or ConSOSL.UFO.664 proteins and to reduce nonspecific background binding, the same two fluorescent labels were conjugated to both proteins. For both time points, we selected ConM-BG505V1V2 SOSIP.v7 negative (−), ConM SOSIP.v7/ConSOSL.UFO.664 positive (+) B cells for single cell sorting. For the second time point we also sorted a selection of ConM-BG505V1V2+, ConM SOSIP.v7/ConSOSL.UFO.664+B cells (Fig. 2a). On average, 0.3% of the total B cell population was reactive to ConM SOSIP.v7 and/or ConSOSL.UFO.664 proteins. The majority of sorted antigen-specific B cells expressed immunoglobulin (Ig) G (69%), while a lower proportion expressed IgM (18%). These frequencies were comparable to the IgG+ and IgM+ frequencies within the total B cells (63% IgG+ and 21% IgM+). Furthermore, we observed a 341-fold enrichment of V1V2-targeting B cells in the sorted fraction (82%), compared to the general total B cell (unsorted) population (0.24%).

We next amplified the B cell receptor (BCR) sequences from the sorted antigen-specific B cells. In total, we obtained 148 antibody heavy chain (HC)/light chain (LC) pairs that were used to generate mAbs. Based on screening for antigen binding by enzyme-linked immunosorbent assay (ELISA), we selected 40 mAbs with high reactivity to ConM SOSIP.v7 and/or ConSOSL.UFO.664 proteins, 18 from the first and 22 from second time point, for further characterization (Fig. 2b). To support the analyses of antigen-specific BCR sequences, we generated individualized HC germline V-gene databases for each animal by deep sequencing pre-immunization PBMCs and applying IgDiscover^31^. This enabled accurate gene assignment, determination of somatic hypermutation (SHM) levels and allowed us to make comparisons to the naive repertoire for each individual animal. Overall, we noted that the majority of mAbs utilized HC V-genes from the IGVH3 family (45%), followed by the IGVH4 (34%), IGVH1 (16%) and IGVH5 (5%) families, respectively (Fig. 2c). Compared to the naive repertoire we observed an enrichment of the IGHV1 (from 2.2 to 16%) and IGHV3 (from 38 to 45%) families and a decrease of IGHV4 gene usage (from 50 to 34%) (Fig. 2c). The naive repertoire IGHV gene frequencies are consistent with findings from IgM repertoires of cynomolgus and rhesus macaques, as was recently described by Chernyshev et al.^32^

The average level of SHM in the HC for mAbs derived from week 25 was 5.8% at a nucleotide level (range: 1.3–9.6%), which increased significantly to 8.2% (range: 0.3–13.4%) at week 71, after boosting with ConSOSL.UFO.664 (*p* = 0.0252) (Fig. 2d). Using IgDiscover analysis we identified two clonal Ab families from animals MCM04 and MCM05 (assigned lineages CM04A and CM05A, respectively), consisting of three mAbs each, including ones from both time points (Supplementary Table 1). The SHM in these families increased from 3.7% (CM04A1) and 9.4%/8.1% (CM05A1/CM05A2) at week 25 to 6.8% (CM04A2/CM04A3) and 13.4% (CM05A3) at week 71 (Fig. 2d). Combined, these data illustrate continued affinity maturation facilitated by the extended time interval and the ConSOSL.UFO.664 booster immunizations.

Overall, the antigen-specific mAbs isolated from all NHPs combined had a mean HC complementarity determining region 3 (CDRH3) length of 15.9 amino acids (aa) (range: 8.0–25.0), with no significant differences between the two time points (Fig. 2e). The mean CDRH3 length of the IgM repertoire in various macaque species—including the specific six animals used in this study—was determined at 14.8 aa^32^, indicating that on average the isolated mAbs have slightly longer CDRH3, a characteristic that is shared with human bNAbs.

### A subset of mAbs neutralize the autologous viruses with high potency

**Fig. 3:**
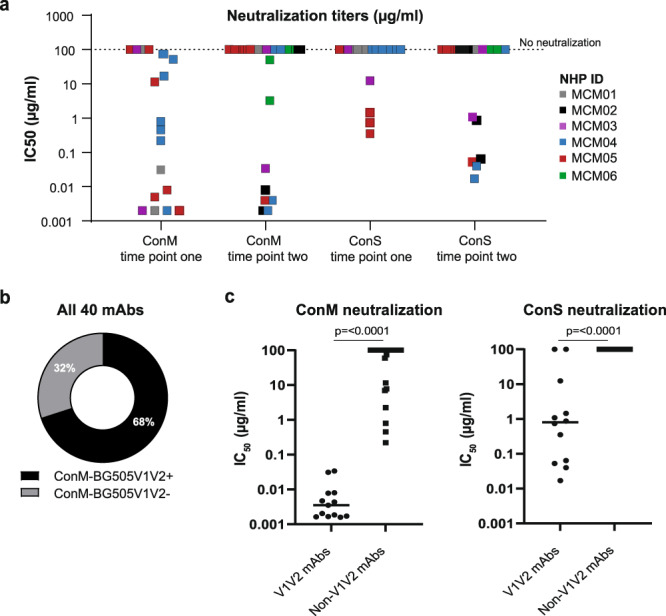
Neutralization specificities of the isolated mAbs. **a** Neutralization ability of all isolated mAbs for ConM and ConS virus, per time point. The y-axis shows the mean IC_50_ values (µg/ml) for each of the mAbs, which are depicted in different colors for each animal. **b** mAbs were differentiated as either V1V2 targeting mAbs (32%) or non-V1V2 targeting mAbs (68%), based on their ability to bind ConM BG505 V1V2 protein in ELISA. **c** Ability of V1V2 targeting and non-V1V2 targeting mAbs to neutralize ConM and ConS virus. The *y*-axes show mean IC_50_ values (µg/ml) for each of the mAbs. V1V2 targeting NAbs were significantly more potent in neutralizing ConM virus (*p* ≤ 0.0001) and ConS virus (*p* ≤ 0.0001). Two-tailed Mann–Whitney U tests were used to determine statistical differences.

Next, we tested the isolated mAbs for their ability to bind and neutralize the autologous ConM virus. All 40 isolated mAbs bound the autologous ConM SOSIP.v7 Env protein in ELISA (Supplementary Table 2), and for each of the NHPs included in this study at least one ConM neutralizing mAb was isolated. At the first time point, 14 out of 18 (78%) isolated mAbs neutralized the ConM virus, with IC_50_ values ranging from 0.002 to 81 µg/ml (Fig. 3a). Ten mAbs showed very potent neutralization with IC_50_ values of <1 µg/ml (Fig. 3a). From the second time point, eight out of 22 (36%) mAbs neutralized the autologous ConM virus (Fig. 3a). Their potencies ranged from 0.002 to 3.7 µg/ml (Fig. 3a).

Generally, (cross-)binding to the ConS Env protein was observed for mAbs isolated before and after the ConSOSL.UFO.664 protein boost (Supplementary Table 2). Interestingly, while none of the week 25 sera neutralized ConS, we observed cross-neutralization of ConS virus by four NAbs, 22.2% of all mAbs isolated at the first time point (Fig. 3a). Their IC_50_ values ranged from 0.35 to 12 µg/ml, showing diverse neutralization potencies (Fig. 3a). The cross-neutralization results suggest that the two consensus sequence Env immunogens share similar immunodominant epitopes. Six mAbs (27%) isolated from the second time point neutralized the ConS virus (Fig. 3a) with potent IC_50_ values ranging from 0.017 to 1.1 µg/ml. Thus, ConS NAbs from week 71 were 11-fold more potent than ConS NAbs from week 25 (median IC_50_ of 0.056 µg/ml versus 1.1 µg/ml) (*p* = 0.11)). The mAbs from both time points that were capable of ConS virus neutralization were, without exception, able to neutralize the autologous ConM virus and consistently neutralized ConM more potently than ConS. These observations suggest that the ConM SOSIP.v7 immunogen initiated the development of these antibody responses and that the ConSOSL.UFO.664 immunogen boosted the pre-existing responses. However, some mAbs only neutralized the ConM virus, suggesting they targeted an epitope that is not present on the ConS virus.

### The majority of NAbs target the V1V2 region

To determine what epitope(s) facilitated cross-neutralization of the two viruses, we studied the ability of isolated mAbs to bind to the ConM-BG505V1V2 protein in ELISA. 13 mAbs (32%) were categorized as V1V2 targeting mAbs, while the remaining 27 mAbs (68%) mAbs were not (Fig. 3b). Nine non-V1V2 mAbs neutralized the ConM virus but not ConS virus (Fig. 3c), seven of which were isolated from the first time point. The other 18 non-V1V2 mAbs were unable to neutralize ConM or ConS. None of the non-V1V2 mAbs neutralized ConS. On the contrary, all 13 V1V2 mAbs neutralized the ConM virus and 10 of these (77%) also (cross-)neutralized the ConS virus (Fig. 3c). These results show that the majority (59%) of NAbs targeted the V1V2 region, consistent with previous studies in rabbits that revealed the V1V2 region as a dominant NAb epitope on ConM SOSIP.v7 trimers^22,28,30^. This region is very similar between the two consensus Envs, with only a 14 aa difference. Furthermore, we found that V1V2 NAbs that neutralized ConM were >500-fold more potent than non-V1V2 NAbs (median IC_50_ of 0.0035 µg/ml versus 2.3 µg/ml) (*p* ≤ 0.0001) (Fig. 3c). The strong autologous serum neutralization titers observed after immunization with consensus immunogens are therefore likely facilitated by NAbs targeting this region of the Env protein.

50% of the isolated NAbs isolated at week 25 were directed to the V1V2 region, whereas this proportion increased to 75% at the second time point (*p* = 0.026). Together with the observations that non-V1V2 region targeting ConM NAbs were isolated primarily at the first time point and did not cross-neutralize ConS, this may indicate that ConSOSL.UFO.664 immunization focused the Ab responses further towards the V1V2 NAb epitope at the expense of other, more broadly neutralizing, epitope specificities.

Three V1V2-targeting NAbs and two non-V1V2 NAbs that were capable of potently neutralizing ConM and ConS virus or ConM virus only were tested against a virus panel representing global HIV-1 diversity to assess their breadth^33^. No neutralization breadth was detected for any of these five NAbs, suggesting that they are specific for the consensus sequences (Supplementary Table 3).

### The immunodominant NAb epitope involves the V3 base

**Fig. 4:**
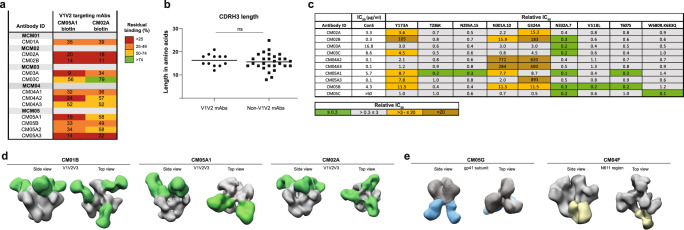
Epitope specification of V1V2 region and gp41 targeting NAbs. **a** Percentage of residual binding of suspected V1V2 targeting NAbs to ConM SOSIP.v7 trimers in presence of a competing biotinylated V1V2 targeting mAb (CM05A1, CM02A). V1V2 targeting mAbs were classified as mAbs that showed competition (<60% residual binding) with either of the two competitor mAbs. **b** Individual CDRH3 amino acid lengths of the V1V2 region targeting and non-V1V2 region targeting isolated mAbs. Two-tailed unpaired t-test was used to determine statistical differences (*p* = 0.2714). **c** Neutralizing activity of 10 NAbs with varying epitopes was measured against a panel of ConS virus mutants. For each mAb, the fold-change of the IC_50_ value against each pseudovirus variant relative to the ConS parental virus is given. This value is indicated as the relative inhibitory concentration 50 (RIC_50_). **d** Negative stain-electron microscopy (NS-EM) was performed on a selection of mAbs to verify various epitope specificities. NS-EM confirmed the binding of CM01B, CM05A1, and CM02A to a region surrounding the V1V2V3 epitope. mAbs are complexed with ConM SOSIP.v9.0 protein. **e** NS-EM confirmed the binding of several NAbs to the gp41 Env subunit, of which CM05G is an example. CM04F also binds the gp41 Env subunit, in a region proximal to the N611 PNGS. mAbs are complexed with ConM SOSIP.v9.0 protein.

To further characterize the immunodominant V1V2 dependent NAb epitope we performed a competition ELISA with two biotinylated V1V2 NAbs isolated in this study, CM05A1 and CM02A (Fig. 4d). All V1V2 NAbs isolated in this study competed with these two biotinylated NAbs (<60% residual binding) (Fig. 4a), suggesting that they all share an overlapping epitope.

The V1V2 region is the target of many potent bNAbs isolated from HIV-1 infected individuals during natural infection in humans, such as PGT145^34^, PG9^35^, and PG16^36^. These V1V2 bNAbs usually have long CDRH3 regions (33, 30, and 30 aa respectively) to penetrate the dense glycan shield surrounding this area of the HIV-1 Env protein. Additionally, they often rely on binding to glycans for their neutralization abilities. We investigated whether the V1V2 targeting NAbs isolated in this study shared similar characteristics, in order to assess whether the elicited responses could have the ability to develop into bNAb responses. The V1V2 targeting mAbs had a mean CDRH3 length of 16 aa (range: 12–21 aa), similar to a mean CDRH3 length of non-V1V2 region targeting mAbs (16 aa, range: 8–25 aa) (Fig. 4b), demonstrating that these NAbs have shorter CDRH3 than bNAbs targeting this region.

Next, we attempted to describe the epitope of these immunodominant and potent NAbs more precisely using neutralization assays with mutant ConS viruses and NS-EM. Firstly, we evaluated the neutralizing activity of 10 NAbs against a panel of ConS virus mutants (Fig. 4c). We observed reduced neutralization ability of all these NAbs against either ConS virus mutants Y173A, N301A, and/or G324A. In contrast the N332A substitution, in effect removing the N332 glycan adjacent to the V3, enhanced neutralization by most NAbs. Other substitutions, including T236K, N295A, V518L, T607S W680R+K683Q had subtle and/or inconsistent effects. Collectively, these data point at an epitope around the V3 base and V1V2 apex involving residue 173 in the V2 region and residues 301 and 324 in the V3 region, with possible involvement of the glycan attached to N301. These findings were corroborated for a sample of eight V1V2 region targeting mAbs by ELISA, using ConM SOSIP.v7 protein mutants containing N301A and G324A mutations, respectively (Supplementary Fig. 3). The enhanced neutralization following removal of the N332-glycan, therefore likely increasing the accessibility to the V3 region, is consistent with an epitope in this area. NS-EM analyses confirmed that three of these mAbs target an epitope at the V1V2V3 region (Fig. 4d). Furthermore, in contrast to V1V2 bNAbs, which bind right at the trimeric symmetry axis and with a 1:1 stoichiometry, the ConM/ConS-specific NAbs bound with a 3:1 stoichiometry targeting an epitope slightly off the symmetry axis. Of note, in particular, CM01B and CM02A appear to target the trimer apex with a more horizontal angle compared to the Fabs detected by the EMPEM analysis (Fig. 1d).

Finally, a series of competition ELISAs were performed using the same biotinylated NAbs (CM05A1 and CM02A) as before and several known V1V2 (PG16, VRC26.25) and N332 (PGT121, PGT125, and PGT128) targeting bNAbs. Competition was steadily observed between the isolated NAbs and PGT121 and PGT125 (51–62% residual binding), confirming a contribution of the V3 base to the epitope. Competition with V1V2 targeting bNAbs however was only inconsistently detected, which could be explained by the difference in angle of approach and stoichiometry as described above (Supplementary Fig. 4).

### Other neutralizing epitopes (non-V1V2V3 region targeting NAbs)

We identified eight neutralizing mAbs that did not target the V1V2V3 region, with potencies ranging from 0.22 to 75 μg/ml. The neutralizing epitopes for these mAbs were specified by NS-EM and competition ELISA. Five NAbs targeted the gp41 subunit of the Env trimer, of which CM05G was imaged as a representative in NS-EM, confirming an epitope in gp41 (Fig. 4e). The non-V1V2V3 NAb CM04F neutralized the ConM virus and targeted the region surrounding the potential N-linked glycosylation site (PNGS) at N611 (Fig. 4e), suggesting that it could accommodate the N611 glycan. For two NAbs, the exact neutralizing epitope could not be determined.

### A subset of non-NAbs targets the trimer base

**Fig. 5:**
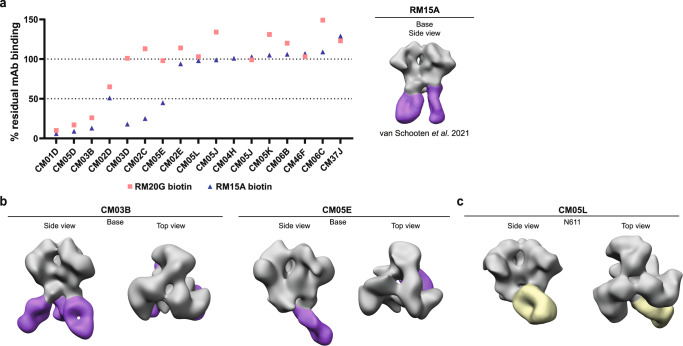
Epitope specification of non-V1V2 region targeting non-NAbs. **a** Percentage of residual binding of suspected non-V1V2 targeting non-NAbs to ConM SOSIP.v7 trimers in presence of a competing biotinylated base binding mAb (RM15A, RM20G). Base binders were classified as mAbs that showed competition (<60% residual binding) with either of the two competitor base binding mAbs. **b**, **c** Negative stain-electron microscopy (NS-EM) was performed on a selection of mAbs to verify various epitope specificities. NS-EM confirmed the binding of CM03B and CM05E to the trimer base and CM04F and CM05L to the N611-glycan region. mAbs are complexed with ConM SOSIP.v9.0 protein.

18 out of 40 mAbs (45%) were unable to neutralize either ConM or ConS virus. Binding to the ConM-BG505V1V2 protein in ELISA indicated that all these non-NAbs target an area on the ConM SOSIP.v7 trimer other than the V1V2V3 region (Fig. 3b). Previous studies have shown that on soluble Env trimer immunogens the exposed trimer base is immunodominant but only induces non-Nabs^24–26^. We studied whether the non-NAbs we identified here targeted the base of the trimer. To this end we performed a binding competition ELISA of the isolated non-NAbs with two previously isolated base binding non-NAbs; RM15A^26^ and RM20G^25^ (Fig. 5a). These known base binding mAbs were isolated from NHPs immunized with soluble BG505 SOSIP.664 Env trimers and are able to cross-bind to ConM SOSIP.v7 protein. Base binders were classified as mAbs that showed competition (<60% residual binding) with either of the two competitor base binding mAbs. Per this definition, seven non-NAbs were categorized as base binders, i.e., 39% of all non-NAbs, 18% of all isolated mAbs (Fig. 5a). These base binders were found at both time points in similar proportions, suggesting that the booster immunization with the ConSOSL.UFO.664 Env trimer did neither reduce nor increase responses to this area. The epitope of these suspected base binders was confirmed by NS-EM (Fig. 5b).

### One non-NAb targets the N611 region

Studying the remaining 10 non-NAbs, NS-EM demonstrated that one mAb, CM05L, targeted a region surrounding the PNGS at N611 (Fig. 5c). This was consistent with the EMPEM analysis (Fig. 1d) which showed that this epitope was often targeted in these NHPs. In BG505 SOSIP protein immunized NHPs this region is often targeted by antibodies as a result of under-occupancy of the N611 PNGS and this suggests that the N611 PNGS might have been under-occupied on the ConM SOSIP.v7 and ConSOSL.UFO.664 trimers used in this study, but not on the corresponding virus as these mAbs did not neutralize the virus.

## Discussion

Artificial consensus Env trimers are designed to minimize clade- or isolate-specific antigenic regions. They incorporate the most common amino acid on each position and thereby limit the presence of unique characteristics. Even though these sequences are not naturally circulating, they yield accurately folded native-like Env trimers. Both Consensus M and Consensus S described here should not contain any strain-specific holes in the glycan shield and do not encode for any rare amino acids in their sequences. Because of these characteristics, consensus Env trimers might have the potential to elicit a more broadly neutralizing antibody response.

Rabbit immunization studies with ConM SOSIP.v7^22^ and ConSOSL.UFO.664^20^ proteins resulted in very strong autologous neutralization titers after three immunizations. Interestingly, these studies show reciprocal cross-neutralization of ConM/ConS viruses, indicating that a shared epitope might be presented by both Env proteins, that is targeted by cross-neutralizing Abs. These studies and others have mapped that particular strain-specific, yet shared immunodominant epitope for ConM SOSIP.v7 and ConSOSL.UFO.664 immunogens to the V1V2 loop region on the apex of the Env trimers^20,22,28^. Other targeted regions have been identified by EMPEM analysis and include the CD4bs, N618/N625, N355/N289, and N611-glycan epitopes and the trimer base^28^.

Here, we investigated the antibody responses in NHPs after three immunizations with ConM SOSIP.v7 immunogen and subsequently after two ConSOSL.UFO.664 immunogen boosts. By isolating mAbs from two time points, we examined the development of antibody responses over time. We report very potent autologous ConM serum neutralization at both time points, and moderate autologous ConS neutralization after the two ConSOSL.UFO.664 protein boosts. By using two different immunogens, we observed that existing responses generated against ConM SOSIP.v7 protein were boosted by ConSOSL.UFO.664 protein, as levels of SHM increased over time in two specific Ab lineages and the very potent neutralizing antibody responses became more focused towards the previously identified immunodominant V1V2 epitope^22,28^. However, in addition to previous findings, the immunodominant epitope appears to extend towards the V3 region.

The isolated NAbs targeting the V1V2V3 epitope were very potent, but did not have long HCDR3s, which are typical of bNAbs directed to the trimer apex^34–36^. This might be attributed to the short V1 loops of the consensus sequences, which allow binding by antibodies with average length CDRH3s. Therefore, one approach to promote the elicitation of more broadly neutralizing antibodies is to extend the V1 region of the consensus sequences as this may select for NAb precursors with longer CDRH3s that may have a greater potential to develop into bNAbs.

Base binding mAbs usually bind a wide array of soluble Env trimers, as the base of the trimer is quite conserved and it is covered by glycans in its natural transmembrane-bound configuration. These Abs are not a desired response as they do not possess neutralizing capacities and might distract the immune response from focusing on the development of (b)NAbs. We, therefore, designed a method to minimize the number of base binding Abs in the initial single B cell sort. We report that roughly 20% of the produced mAbs are base binders. In comparison, other studies have reported the proportions of base binders to be up to 66% of isolated mAbs^25,26^. This shows that negative selection in the initial stages of mAb generation help exclude undesired mAbs. Nonetheless, it remains relevant to know the proportion of base binding mAbs as a fraction of the total Ab response. Another way to determine this is to visualize all the dominant antibody targets by EMPEM analysis.

While the rationale behind the use of consensus immunogens was to minimize strain-specific responses and improve antibody breadth, a new yet artificial strain-specific immunodominant epitope was unintentionally generated because of several reasons. Firstly, when comparing the V1V2 sequence to global sequences found in the Los Alamos National Laboratory (LANL) HIV databases (www.hiv.lanl.gov), we found that less than five percent of the sequences have a four aa stretch overlap with the Consensus M V1V2 loops (Supplementary Fig. 5a). Secondly, the consensus Env proteins have relatively short V1V2 regions (Supplementary Fig. 5b). The Consensus M and Consensus S sequences used in this study have V1 loops of 21 and 24 aa, and V2 loops of 40 and 44 aa, respectively. Lastly, even though PNGS are present at a similar frequency as in natural isolates (Supplementary Fig. 5b), the PNGS that are present in the V1V2 region are in fact largely under occupied (Supplementary Fig. 5c), exposing an immunodominant epitope. Considering the results of this study it is evident that consensus Env designs, like non-consensus Env designs, will need further optimization to shield *de novo* strain-specific epitopes to improve the antibody responses. Although the current consensus sequences are an average of the globally circulating HIV-1 viruses, the resulting Env proteins still contain too many unique characteristics to induce the desired bNAb response. It is therefore plausible that engineering consensus sequences with longer and/or more glycosylated V1V2V3 loops, as well as shielding of the Env base, i.e., by the expression on nanoparticles, might redirect the antibody response to epitopes more conducive to inducing neutralization breadth, increasing the potential of consensus sequences in the use for HIV-1 vaccine design.

We have shown here in preclinical studies that the NAb responses induced by native-like consensus immunogens are dominated by specificities targeting the V1V2V3 domains. Potent NAb responses targeting the V1V2 region were also observed in NHP immunization studies using 16055-based native flexibly linked (NFL) trimers^37,38^. It remains to be seen whether the V1V2V3 region is also predominantly targeted by NAbs in humans, as data from early-stage clinical trials become available (ClinicalTrials.gov Identifiers: NCT03816137, NCT03961438, and NCT04046978). If so, the data assembled here will help to interpret these responses and accelerate the use and improvement of consensus-based proteins. Such improved consensus-based Env proteins could for example be used to boost immune responses as so called ‘polishing’ immunogens. These immunogens are designed to further shape the Ab response to recognize bNAb epitopes with high affinity to diverse HIV Env proteins, following priming with a germline targeting protein such as BG505 SOSIP.GT1.1^39^, aimed to activate specific B cell germline precursors of bNAbs. Like ConM SOSIP.v7, BG505 SOSIP.GT1.1 is currently under evaluation in a phase 1 clinical trial (ClinicalTrials.gov Identifier NCT04224701), paving the way for (clinically) evaluating germline-targeting following by a polishing of the response by consensus-based immunogens in humans.

## Methods

### Ethics statement

Cynomolgus macaques (*Macaca fascicularis*) originating from Mauritius and imported from AAALAC certified breeding centers were used in this study. All animals were housed in groups at the IDMIT infrastructure facilities (CEA, Fontenay-aux-roses, Animal facility authorization #D92-032-02, Prefecture des Hauts de Seine, France) and in compliance with European Directive 2010/63/EU, the French regulations, and the Standards for Humane Care and Use of Laboratory Animals, of the Office for Laboratory Animal Welfare (OLAW, assurance number #A5826-01, US). The protocols were approved by the institutional ethical committee “Comité d’Ethique en Expérimentation Animale du Commissariat à l’Energie Atomique et aux Energies Alternatives » (CEtEA #44) under statement number A15-073. The study was authorized by the “Research, Innovation and Education Ministry” under registration number APAFIS#3132-2015121014521340.

### Processing of animal products

Six adult, female cynomolgus macaques, aged 33 to 42 months were immunized on week 0 (W0), W8, and W24 with 20 µg ConM SOSIP.v7 and subsequently on W38 and W70 with 20 µg ConSOSL.UFO.664. ConM SOSIP.v7 was administered to MCM01, MCM02, and MCM03 adjuvanted with a liposomal formulation of monophosphoryl lipid A (MPLA), intramuscularly (IM). MCM05 and MCM06 received ConM SOSIP.v7 adjuvanted with MPLA, subcutaneously (SC). Lastly for MCM04 ConM SOSIP.v7 was adjuvanted with squalene emulsion (SE) and administered IM. All vaccinations with ConSOSL.UFO.664 were adjuvanted with liposomal MPLA and administered IM. Blood was taken at 25 and 71 weeks after the first immunization for peripheral blood mononuclear cell (PBMC) isolation and on weeks 26 and 72 for serum sampling. PBMCs were isolated from the blood using Ficoll as previously described^40^. In short, phosphate-buffered saline (PBS) diluted blood was loaded on a Ficoll layer and centrifuged for 30 min at room temperature (RT) at 4000 × *g* with a deceleration speed of 0. From this PBMCs were isolated, washed with PBS and re-suspended in ACK buffer (Thermo Fisher Scientific). PBMCs were subsequently suspended in 10% DMSO in fetal calf serum (FCS) and frozen at −150 °C.

### Sorting of ConM SOSIP.v7/ConSOSL.UFO.664 immunogen reactive B cells

Frozen PBMCs were thawed and washed with PBS to use for fluorescently labeled cell sorting (FACS) to select for ConM SOSIP.v7 and ConSOSL.UFO.664 specific B cells. 2 µg biotinylated ConM SOSIP.v7 and ConSOSL.UFO.664 Env proteins were both conjugated to both 0.5 mg streptavidin-AF647 and 0.25 mg streptavidin-BV421 (BioLegend). Biotinylated ConM-BG505V1V2 Env protein was conjugated to 0.25 mg streptavidin-BB515 (BD Horizon). ~10 million PBMCs were stained with all conjugated ConM SOSIP.v7, ConSOSL.UFO.664, and ConM-BG505V1V2 Env proteins, Via eF780 (Invitrogen), IgM BV605 (BioLegend), IgG PE-cy7 (BD Horizon), CD20 PE-CF594 (BD Horizon), and CD27 PE (BD Horizon). Staining was done for 30 min at 4 °C in the dark. Subsequently, cells were washed twice and re-suspended in PBS+FCS (1:100). Cells were analyzed and single cell sorted using the FACS Aria III cell sorter (BD Biosciences) using index sorting. The gating strategy used is depicted in Supplementary Fig. 6. We first selected lymphocytes and single cells, then life CD20+B cells. Probe binding was analyzed within this CD20+B cell population. Immunoglobulin (Ig) subtype and CD27 expression was measured. Env probe specific B cells were single cell sorted in a 96-wells plate containing lysis buffer (RNAse inhibitor (40 U/µl) (Thermo Fisher Scientific), 5X First strand superscript III buffer (Invitrogen), 0.1 M dithiothreitol (DTT) (Invitrogen), and Milli-Q) and immediately stored at −80 °C.

### Amplification and cloning of antibody variable regions

mRNA of the lysed and sorted B cells was converted to cDNA by a reverse transcriptase (RT)-PCR reaction^40^. 6 µL RT-PCR reaction mixture (random hex primers 200 ng/µL (Thermo Fisher Scientific), dNTP mix 6 mM each (New England BioLabs), 50 U Superscript III RTase, and MQ) was added directly to the lysed and sorted B cells. The following PCR program was run; 42 °C, 10 min 25 °C, 10 min 50 °C, 60 min 95 °C, 5 min. cDNA was subsequently used to amplify DNA of the heavy and light (kappa and lambda) chain of the expressed B cells in three PCR reactions for each chain. For the heavy chain: PCR 1 used HotStar Plus DNA polymerase (0.38 U) (Qiagen) in a mixture of 10× PCR buffer, dNTPs (New England BioLabs) (0.2 mM), forward and reverse primer sets^25^ (0.17 µM), 2 µl of RT-PCR cDNA and MQ. PCR program run: 95 °C, 5 min; 50 cycles of 94 °C 30 s, 55 °C, 30 s, 72 °C, 1 min. PCR 2 was done with MyTaq polymerase (0.5 U) (Bioline) using 2 µl of PCR 1 DNA product, 5× MyTaq reaction buffer, forward and reverse primer sets (0.1 µM) and MQ in a total volume of 20 µl. PCR program run: 95 °C, 1 min; 50 cycles of 95 °C, 15 s; 55 °C, 15 s; 72 °C, 45 s. The final PCR was used to create the overhang needed for the subsequent cloning reactions. This PCR 3 was done with HotStar HiFi DNA polymerase (0.25 U) (Qiagen) in a mixture of 5x PCR buffer, forward and reverse primer mixes (1.3 µM), MQ, and 2 µl DNA product from PCR 2. For both kappa and lambda light chains PCR 1 and PCR 2 were done using MyTaq DNA polymerase (Bioline) (0.25 U) as described for the heavy chain, using kappa or lambda primer sets (1.3 µM) and 2 µl of either RT-PCR or PCR 1 DNA product. Annealing temperatures for kappa chain PCR 1 were adapted to 56 °C and for PCR 2 to 55 °C. For the lambda chain the annealing temperatures were 52 °C and 53 °C for PCR 1 and PCR 2 respectively. PCR 3 for both kappa and lambda chains was done with HotStar Plus DNA polymerase (0.38 U) (Qiagen) in a mixture of 10× PCR buffer, dNTPs (0.2 mM), specific forward and reverse primer sets (0.17 µM), MQ, and 2 µl of PCR 2 DNA product. Following PCR program was run: 95 °C, 1 min; 25 cycles of 94 °C, 30 s; 58 °C, 30 s; 72 °C, 1 min.

PCR 3 DNA product was used for Gibson cloning into expression vectors^41^. 1 µl PCR product was added to 1 µl of expression vector DNA and 2 µl of 2× Gibson mix (T5 exonuclease (0.2 U) (Epibio), Phusion polymerase (12.5 U) (New England BioLabs), Taq DNA ligase (2000 U) (New England BioLabs), Gibson reaction buffer (0.5 g PEG-8000, 1 M Tris/ HCl pH 7.5, 1 M MgCl_2_, 1 M DTT, 100 mM dNTPs, 50 mM NAD (New England BioLabs), MQ). Mixtures were incubated for 1 h at 50 °C, after which they were transformed into competent XL-1 blue cells (Agilent) for mini- or midiprep DNA isolation using the Macherey-Nagel plasmid DNA isolation kit.

### Envelope glycoprotein design and production

For single B cell sorting and subsequent analysis the ConM SOSIP.v7 Env protein, ConM-BG505V1V2 variant, and ConSOSL.UFO.664 protein were used. These Env proteins were produced using PGT145 affinity columns^20,22^. The expression plasmids of the protein and furin were transiently expressed (ratio 4:1) in HEK293F cells (Invitrogen, catalog number R79009). The Env trimers were harvested from the cell supernatants at day 7 by centrifugation at 4000 × *g* for 20 min, followed by 0.22 µM steritop- vacuum-filteration before purification by gravity-driven chromatography on a PGT145 antibody-conjugated Sepharose column. Env proteins were eluted with 3 M Mg_2_Cl_2_ pH 7.8, directly into neutralization buffer (20 mM TrisHCl pH8.0, 75 mM NaCl). After purification, Env proteins were concentrated and buffer exchanged into phosphate-buffered saline (TBS) to a final concentration of >0.5 mg/ml using Vivaspin 100kD filters (GE healthcare). Purified ConM SOSIP.v7 and ConSOSL.UFO.664 Env trimers were biotinylated for FACS using the BirA biotin protein ligase kit (Avidity) following the manufacturer’s instructions. In addition, ConM SOSIP.v9.0 and ConS SOSIP.v9.0 proteins were produced in a similar way and used for antibody analyses^42^.

ConM SOSIP.v9.0 trimers were analyzed using SDS-PAGE and BN-PAGE followed by Coomassie blue staining and thermal denaturation was studied using nano-differential scanning calorimetry (DSC)^22^. In short, Env trimers were first buffer exchanged to PBS, and concentration was adjusted to ~0.25 mg/mL. After loading the sample into the cell of a nano-DSC calorimeter (TA instruments, The Netherlands), thermal denaturation was probed at a scan rate of 60 °C/h. Buffer correction, normalization, and baseline subtraction procedures were applied before the data were analyzed using the NanoAnalyze Software v.3.3.0 (TA Instruments). The data were fitted using a non-two-state model, as the asymmetry of some of the peaks suggested that unfolding intermediates were present. Trimer morphology was assessed by NS-EM, as described below.

### Antibody production and purification

To express mAbs for characterization, heavy and light chain plasmid DNA was co-transfected into HEK293F cells and purified (Invitrogen, catalog number R79009)^19,40^. 40 µg of each heavy and light chain DNA was added with 240 µg PEImax transfection agent to OptiMEM. Mixture was incubated for 20 min at RT, poured to HEK293F cells, and incubated for 5 days at 37 °C, shaking. Supernatant was harvested by spinning down the cells for 30 min at 4000 × *g* and subsequently was 0.22 µM filtered using vacuum. Expressed mAbs were affinity purified using Protein G (Thermo Fisher Scientific) coupled beads. Protein G bound mAbs were eluted using 0.1 M glycine, pH 2.0. Eluted mAbs were concentrated to a final volume of 200–500 µl using Vivaspin filters with a cut-off value of 100 kDa (Cytiva). Isolated mAbs were named according to animal identification number and lineage. Sequences were uploaded to GenBank.

mAbs were biotinylated for competition ELISA. Biotinylation was done using the EZ-Link™ Sulfo-NHS-LC-Biotinylation Kit (Thermo Fisher Scientific) following the manufacturer’s protocol. For NS-EM analysis, polyhistidine (His)-tagged Fabs were produced by Gibson cloning Ab heavy chain PCR inserts into a digested Fab expression vector. Gibson reaction products were transformed into competent XL-1 blue cells (Agilent) for mini- or midiprep DNA isolation using the Macherey-Nagel plasmid DNA isolation kit. His-tagged Fabs were recombinantly expressed and secreted as a soluble protein in HEK293F cells. The supernatant was concentrated and loaded onto a Ni-NTA affinity column, and the Fabs were eluted using an imidazole gradient. Fabs were concentrated and buffer exchanged into Tris-buffered saline (TBS) buffer (50 mM Tris, 150 mM NaCl, pH 7.5) using Vivaspin filters with a cut-off value of 10 kDa (Cytiva).

### TZM-bl Neutralization assays

TZM-bl cell neutralization assays using Env-pseudotyped viruses were performed at three sites: the Amsterdam UMC (AUMC)^22^, Duke University (DU)^43,44^ and Ospedale San Raffaele(OSR)^45^. Neutralization by Abs and sera were measured as a function of reductions in luciferase (Luc) reporter gene expression after a single round of infection in TZM-bl cells^43–45^. At the AUMC, mAb dilution (start concentrations ranging from 1 to 100 µg/ml) series and virus were incubated for 1 h at RT and subsequently added to TZM-bl reporter cells. After 3 days infectivity was measured. IC_50_ values were determined as the concentration at which 50% of the infectivity was inhibited. At Duke University, a pre-titrated dose of virus was incubated with serial 3-fold dilutions of heat-inactivated (56 °C, 30 min) serum samples in duplicate in a total volume of 150 μl for 1 h at 37 °C in 96-well flat-bottom culture plates. Freshly trypsinized cells ((10,000 cells in 100 μl (DU) or 75 μl (OSR) of growth medium (GM) containing 75 μg/ml (DU) or 45 μg/ml (OSR) DEAE dextran) were added to each well. One set of control wells received cells + virus (virus control) and another set received cells only (background control). At OSR, after 48 h of incubation, the medium was removed and 50 μl of Brite-Glo reagent (Promega, Madison, Wisconsin, USA) diluted 1:2 with GM was dispensed into each well. The plate was incubated at room temperature for 2 min to allow complete cell lysis. 40 μl was transferred to a corresponding 96-well white plate and analyzed in a luminometer (Mithras (Berthold, Germany)). At DU, after 48 hours of incubation, 100 µl of cells was transferred to a 96-well black solid plate (Costar) for measurements of luminescence using the Britelite Luminescence Reporter Gene Assay System (PerkinElmer Life Sciences). Neutralization titers are the dilution (serum/plasma samples) or concentration (mAbs) at which relative luminescence units (RLU) were reduced by 50% or 80% compared to virus control wells after subtraction of background RLUs. Assay stocks of molecularly cloned Env-pseudotyped viruses were prepared by transfection in 293T/17 cells (American Type Culture Collection) and titrated in TZM-bl cells as described^43,44^. This assay has been formally optimized and validated^46^ and was performed in compliance with Good Clinical Laboratory Practices, including participation in a formal proficiency testing program^47^. Additional information on the assay and all supporting protocols may be found at: http://www.hiv.lanl.gov/content/nab-reference-strains/html/home.htm.

### Negative stain electron microscopy of monoclonal antibodies

ConM SOSIP.v9.0/ConS SOSIP.v9.0 and Fab/IgG complexes were made by mixing 3 µg of ConM SOSIP.v9.0 or ConS SOSIP.v9.0 trimers with 6-fold molar excess monoclonal Fabs or two-fold molar excess monoclonal IgGs. Complexes were incubated for 30 min at RT before being diluted to 30 ng/µl in Tris-buffered saline (TBS). Samples were applied to glow discharged carbon-coated Cu400 EM grids and blotted after 10 s. 3 µl of 2% (w/v) uranyl formate was then applied and immediately blotted. After 10 s, 3 µl of uranyl formate was applied and after 45 s a final blot was performed. Image collection was performed on either a FEI Tecnai TF20 microscope (1.77 Å/pixel; 62,000× magnification) or Tecnai Spirit microscope (2.06 Å/pixel; 52,000× magnification) operating at 120 keV using Leginon^48^. 2D and 3D classification and sorting were performed using Relion v3.0^49^ and electron density maps were visualized in UCSF Chimera^50^.

### Negative stain electron microscopy of polyclonal antibodies

Electron-microscopy-based epitope mapping (EMPEM) experiments were performed^27^. IgGs were purified from serum of immunized animals using protein G resin (Thermo Scientific), at a ratio of 2 ml diluted resin for each ml of undiluted plasma or serum. Samples were diluted at least 4-fold in PBS, then incubated with protein G resin overnight at 4 °C. The resin was washed with 3 column volumes of PBS, and the IgGs eluted with 9 ml of 0.1 M glycine pH 2.5, immediately neutralized with 1 ml 1 M Tris-HCL pH 8. Buffer was exchanged to PBS by centrifugation using 100 kDa Vivaspin 6 column filters with a cut-off value of 100 kDa (Cytiva).

Purified polyclonal IgGs were first digested to Fabs by activating papain for 15 min at 37 °C in digestion buffer (100 mM Tris pH 8, 2 mM EDTA, 10 mM L-cysteine). IgG samples were then digested for four hours at 37 °C with freshly-activated papain and the digestion reactions were stopped using 50 mM iodoacetamide. Digested IgG samples were buffer exchanged into TBS using Amicon ultrafiltration units with a 10 kDa cutoff (EMD Millipore Sigma), and Fabs were further purified by size exclusion chromatography (Superdex 200 Increase 10/300 gl column running in TBS). Fractions corresponding to Fab peaks were pooled and concentrated with Amicon ultrafiltration units with a 10 kDa cutoff (EMD Millipore Sigma). The Fab-SOSIP complexes were assembled using 300 µg of Fab and 15 µg of ConM SOSIP.v9.0 or ConS SOSIP.v9.0 for 18 h at RT. The Fab-SOSIP complexes were purified using size exclusion chromatography (Superose 6 Increase 10/300 gl column running in TBS), and concentrated with Amicon ultrafiltration units with a 10 kDa cutoff (EMD Millipore Sigma).

The purified Fab-SOSIP complexes were diluted to 50 µg/ml in TBS and 3 µl was applied to glow discharged carbon-coated Cu400 EM grids and blotted after 10 s. To stain the complexes, 3 µl of 2% (w/v) uranyl formate was applied and immediately blotted. After 10 s, 3 µl of uranyl formate was applied again and blotted after 45 s. Data were collected on either a Tecnai TF20 electron microscope (FEI) (200 kV, 62,000× magnification) equipped with a TemCam F416 CMOS (TVIPS) camera, or a Tecnai Spirit (FEI) (120 kV, 52,000× magnification) equipped with an Eagle 4 K CCD (FEI/Thermo Fisher) camera. The Leginon automated interface^48^ was used to acquire the data.

Data processing was performed as follows. Particles were auto-picked and extracted using Appion data processing suite^49^. 2D classification was performed using Relion v3.0^51^ into 100 classes, and classes containing Fab-SOSIP complexes were selected for 3D analysis. Particles from the selected classes were 3D sorted into 40 classes using Relion v3.0, with a low-resolution model of unliganded HIV Env as a reference. The 3D maps were visualized using UCSF Chimera^50^. Particles from similar-looking classes were then selected and reclassified using Relion v3.0. The final 3D maps were visualized and segmented using UCSF Chimera^50^ and Segger^52^, respectively. Representative reconstructions (all specificities for a particular animal and time point) have been deposited to the Electron Microscopy Data Bank (EMDB) (see “Data availability”) and summarized in Supplementary Table 4. Additional maps can be requested from the corresponding author.

### Site-specific glycan analysis using mass spectrometry

Three aliquots of ConM SOSIP v7 protein were denatured for 1 h in 50 mM Tris/HCl, pH 8.0 containing 6 M of urea and 5 mM DTT. Next, Env proteins were reduced and alkylated by adding 20 mM iodoacetamide (IAA) and incubated for 1 h in the dark, followed by a 1 h incubation with 20 mM DTT to eliminate residual IAA. The alkylated Env proteins were buffer-exchanged into 50 mM Tris/HCl, pH 8.0 using Vivaspin columns (3 kDa) and two of the aliquots were digested separately overnight using trypsin, chymotrypsin (Mass Spectrometry Grade, Promega) or alpha lytic protease (Sigma Aldrich) at a ratio of 1:30 (w/w). The next day, the peptides were dried and extracted using C18 Zip-tip (MerckMilipore). The peptides were dried again, re-suspended in 0.1% formic acid and analyzed by nanoLC-ESI MS with an Ultimate 3000 HPLC (Thermo Fisher Scientific) system coupled to an Orbitrap Eclipse mass spectrometer (Thermo Fisher Scientific) using stepped higher energy collision-induced dissociation (HCD) fragmentation. Peptides were separated using an EasySpray PepMap RSLC C18 column (75 µm × 75 cm). A trapping column (PepMap 100 C18 3 μM 75 μM × 2 cm) was used in line with the LC prior to separation with the analytical column. The LC conditions were as follows: 280 min linear gradient consisting of 4–32% acetonitrile in 0.1% formic acid over 260 min followed by 20 min of alternating 76% acetonitrile in 0.1% formic acid and 4% Acn in 0.1% formic acid, used to ensure all the sample had eluted from the column. The flow rate was set to 300 nL/min. The spray voltage was set to 2.7 kV and the temperature of the heated capillary was set to 40 °C. The ion transfer tube temperature was set to 275 °C. The scan range was 375–1500 m/z. Stepped HCD collision energy was set to 15, 25, and 45% and the MS2 for each energy was combined. Precursor and fragment detection were performed using an Orbitrap at a resolution MS1 = 120,000. MS2 = 30,000. The AGC target for MS1 was set to standard and injection time set to auto which involves the system setting the two parameters to maximize sensitivity while maintaining cycle time.

Glycopeptide fragmentation data were extracted from the raw file using Byos (Version 3.5; Protein Metrics Inc.). The glycopeptide fragmentation data were evaluated manually for each glycopeptide; the peptide was scored as true-positive when the correct b and y fragment ions were observed along with oxonium ions corresponding to the glycan identified. The MS data was searched using the Protein Metrics 305 N-glycan library with sulfated glycans added manually. The relative amounts of each glycan at each site as well as the unoccupied proportion were determined by comparing the extracted chromatographic areas for different glycotypes with an identical peptide sequence. All charge states for a single glycopeptide were summed. The precursor mass tolerance was set at 4 and 10 ppm for fragments. A 1% false discovery rate (FDR) was applied. The relative amounts of each glycan at each site as well as the unoccupied proportion were determined by comparing the extracted ion chromatographic areas for different glycopeptides with an identical peptide sequence. Glycans were categorized according to the composition detected.

HexNAc(2)Hex(10+) was defined as M9Glc, HexNAc(2)Hex(9–5) was classified as M9 to M3. Any of these structures containing a fucose were categorized as FM (fucosylated mannose). HexNAc(3)Hex(5–6)X was classified as Hybrid with HexNAc(3)Hex(5-6)Fuc(1)X classified as Fhybrid. Complex-type glycans were classified according to the number of HexNAc subunits and the presence or absence of fucosylation. These compositions were further grouped into high mannose (oligomannose and hybrid-type), complex and unoccupied. Two analytical repeats were performed with this method

### Site-specific analysis of low abundance N-glycan sites using mass spectrometry

To obtain data for sites that frequently present low intensity glycopeptide the glycans present on the glycopeptides were homogenized to boost the intensity of these peptides. This analysis loses fine processing information but enables the ratio of oligomannose: complex: unoccupied to be determined. The remaining glycopeptides were first digested with Endo H (New England BioLabs) to deplete oligomannose- and hybrid-type glycans and leave a single GlcNAc residue at the corresponding site. The reaction mixture was then dried completely and resuspended in a mixture containing 50 mM ammonium bicarbonate and PNGase F (New England BioLabs) using only H_2_O18 (Sigma-Aldrich) throughout. This second reaction cleaves the remaining complex-type glycans but leaves the GlcNAc residues remaining after Endo H cleavage intact. The use of H_2_O18 in this reaction enables complex glycan sites to be differentiated from unoccupied glycan sites as the hydrolysis of the glycosidic bond by PNGaseF leaves a heavy oxygen isotope on the resulting aspartic acid residue. The resultant peptides were purified as outlined above and subjected to reverse-phase (RP) nanoLC-MS. Instead of the extensive N-glycan library used above, two modifications were searched for: +203 Da corresponding to a single GlcNAc, a remnant of an oligomannose/hybrid glycan, and +3 Da corresponding to the O18 deamidation product of a complex glycan. Data analysis was performed as above and the relative amounts of each glycoform determined, including unoccupied peptides. The data for the two methods was combined to optimize glycan site coverage of the sample.

### V-gene and SMH determination through IgDiscover

Individualized germline IGHV gene databases from NHP MCM01, MCM02, MCM03, MCM04, MCM05, and MCM06 were produced from samples obtained before immunization. In brief, IgM libraries for deep sequencing of antibody repertoires were generated with 5′ multiplex PCR from total PBMC mRNA^53^. The mRNA was reverse transcribed with IgM constant region-specific primer containing a unique molecular identifier and a universal outer primer sequence. The cDNA was amplified utilizing the universal 3′ primer and two primer sets covering all gene families and targeting leader and UTR respectively that yield two libraries for each animal. Illumina indices and adapters were introduced by PCR prior to sequencing the library with Illumina’s MiSeq v3 kit. The output library was analyzed with IgDiscover to infer the germline gene IGHV alleles^31^. The IGHV database utilized as an input database for the IgDiscover analysis was obtained from genomic DNA sequencing^54^, with the addition of non-located (NL) alleles inferred from a larger set of NHPs^53^. The heavy chain sequences of the isolated mAbs were assigned to the IGHV individualized database from the respective NHP and a comprehensive IGHJ database obtained from a larger group of NHPs, whereas the light chain was assigned to the IMGT’s light chain database using IgBlast to obtain the SHM and CDR3 sequences.

### Binding and competitive ELISAs

Binding and competitive ELISAs were performed using *Galanthus Nivalis Lectin* (GNL) coated (in 0.1 M NaHCO_3_ pH 8.6, overnight) high binding half area plates^26^. Subsequently the plates were blocked with casein (Thermo Fisher Scientific) for 30 min. Plates were washed with Tris-buffered saline (TBS) between each of the subsequent steps. Afterward, 2 µg/ml Env protein was added to the plates and incubated for 2 h. Subsequently mAb dilution series were added to the plate at starting concentration of 1 µg/ml to generate binding curves. After 2 h a 1:3000 dilution of horseradish peroxidase labeled (HRP) goat-anti-human (SeraCare 0.1 µg/ml) was added and left for 1 h at RT. Plates were washed with TBS + 0.05% Tween20 and developed using 1% 3,3′,5,5′-tetramethylbenzidine (Sigma-Aldrich), 0.01% H_2_O_2_, 100 mM sodium acetate, and 100 mM citric acid. Reactions were stopped using 0.8 M H_2_S0_4_ and absorption was measured at OD_450_ using the SpectroStar Nano Spectrophotometer (BMG Labtech). The competitive ELISAs were executed using the same protocol with minor adjustments. The primary Ab was added as a single concentration of 10 µg/ml in triplo and incubated for 0.5 h at RT. Without an additional wash step, a biotinylated competitor Ab was added at 3× the effective concentration (EC)70 value and incubated for another 1.5 h at RT. Binding of the competitor mAb was detected using 1:3000 streptavidin-HRP (BioLegend) as a secondary antibody.

### Webalignment and weblogo

The length, the number of PNGS and charge of the V1V2 region (130-197) of all *env* sequences in the 2020 webalignment from the Los Alamos HIV Databases (http://www.hiv.lanl.gov/) were determined using the AnalyzeAlign tool of the Los Alamos HIV Databases. The V1V2 sequence of ConM and the weblogo of the V1V2 region (130–197) of all *env* sequences in the 2020 webalignment were made using http://weblogo.threeplusone.com/create.cgi.

### Statistical analyses

All statistical analyses (two-tailed unpaired t-test and two-tailed Mann–Whitney U test) were performed in Graphpad Prism 9.1.0.

### Reporting summary

Further information on research design is available in the Nature Research Reporting Summary linked to this article.

## Supplementary information

Supplementary Figures and Tables

REPORTING SUMMARY

## Data Availability

Full antibody sequences are available upon request to the corresponding author. The ConM SOSPIP.v7/ ConSOSL.UFO.664-specific BCR sequences are deposited to GenBank with accession numbers OM160776–OM160815. Negative-stain EM 3D reconstructions have been deposited to the Electron Microscopy Data Bank (EMDB) with accession codes EMD-14888–14908, summarized in Supplementary Table 4.
