# Supplementary material for: Fine-mapping the immunodominant antibody epitopes on consensus sequence-based HIV-1 envelope trimer vaccine candidates
Source: NPJ Vaccines. 2022 Nov 25;7:152. doi: 10.1038/s41541-022-00576-9 (PMC9700725; doi:10.1038/s41541-022-00576-9)
Supplement: Supplementary file 1 — Supplementary Figures and Tables [file 41541_2022_576_MOESM1_ESM.pdf]

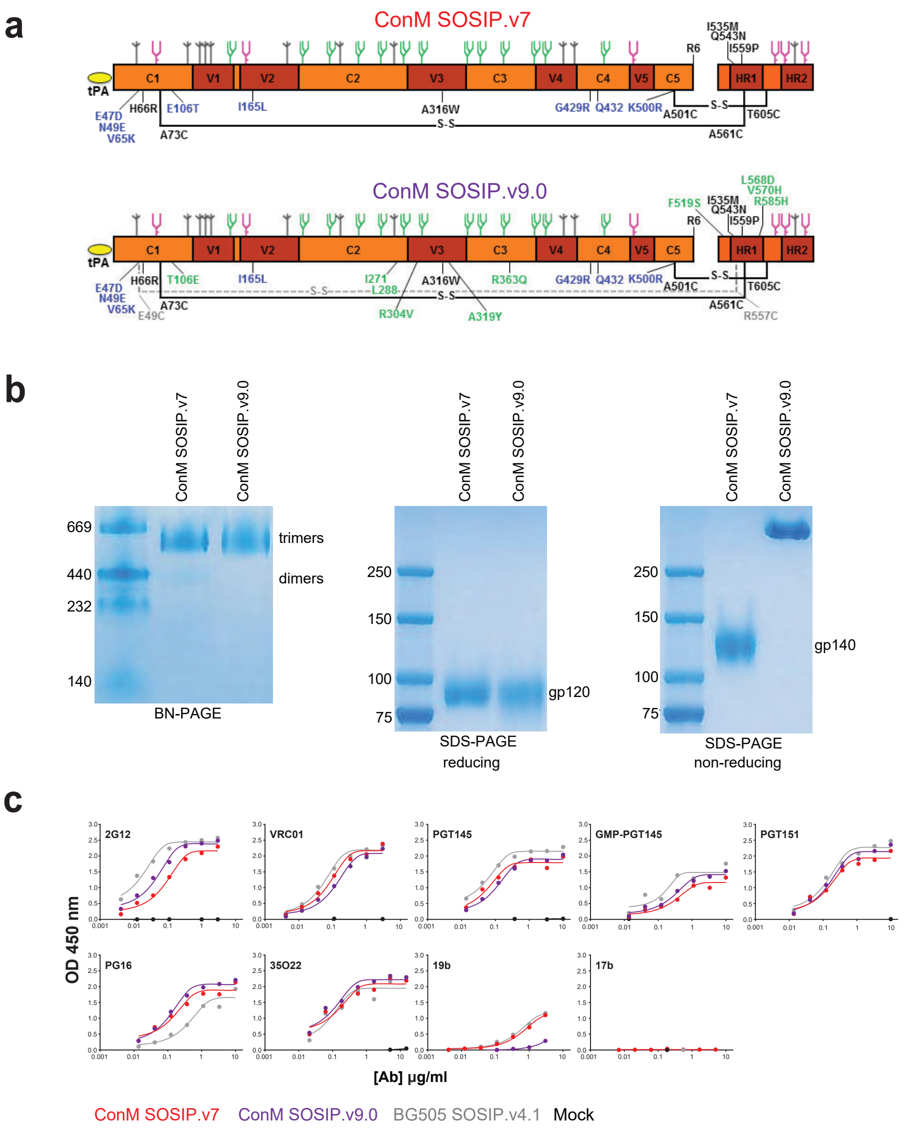

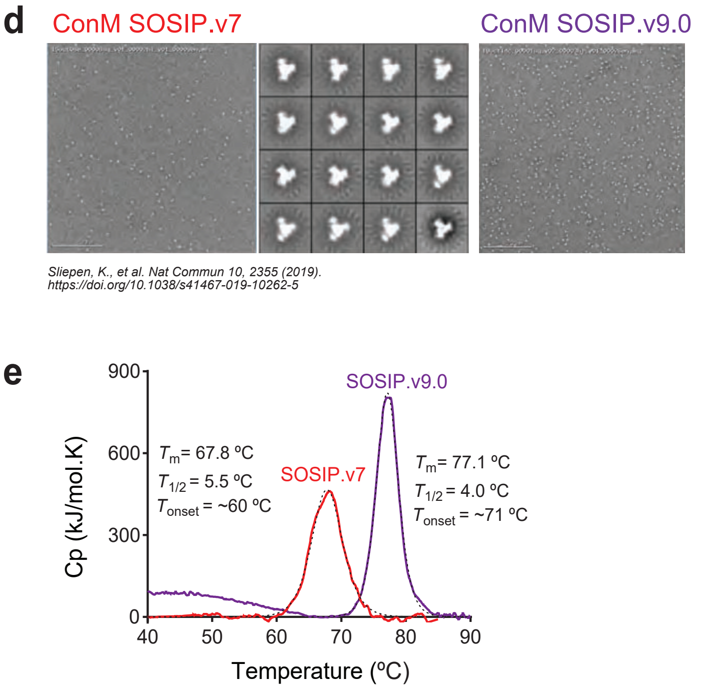

f

	Predicted glycan holes			
ConM SOSIP.v7	N359	N457	N459	N467
ConM SOSIP.v9.0			N459	

g

	Production			Morphology	Thermostability	Glycan composition		
				NS-EM[b]	DSC[b]	HILIC-UPLC[b]		
	Yield[b]	Yield[b]	Yield[b]	Native-like trimers	T_m	Man_8	Man_9	Oligo-mannose
SOSIP version[a]	mg/L	mg/L	mg/L	%	ºC	%	%	%
ConM SOSIP.v7	4.90[c]	3.78[d]	ND	100[c]	67.8[c]	12[c]	25[c]	68[c]
ConM SOSIP.v9.0	6.51[c]	2.17[d]	3.86[e]	100[d]	77.1[d]			

a
b A linear representation and an overview of the modifications made to the trimer variants is shown in figure S1A.
c Data were derived from 293F cell-expressed and PGT145-purified SOSIP trimers.
d Data were derived from SOSIP trimers containing a D7324-tag.
e Data were derived from SOSIP trimers without a tag.
Data were derived from SOSIP trimers containing a HIS-tag.

Supplementary Figure 1. ConM SOSIP.v9.0 trimer characteristics

(**a**) A linear representation and an overview of the modifications made to the version 9.0 trimer variant, compared to version 7. (**b**) ConM SOSIP.v9.0 was analyzed by BN-PAGE analysis, reducing (+DTT) and non-reducing (-DTT) SDS-PAGE followed by Coomassie Blue staining. (**c**) Lectin-capture ELISA with purified ConM SOSIP.v9.0 trimers. (**d**) 2D class averages of negative-stain electron microscopic analyses of purified ConM SOSIP.v722 and ConM SOSIP.v9.0 trimers. (**e**) Thermostability derived from differential scanning calorimetry (DSC) of purified ConM SOSIP.v7 and ConM SOSIP.v9.0 trimers. The DSC parameters midpoint of thermal transition (*T*m), starting temperature of unfolding (*T*onset), and width of the transition at half peak height ($T_{1/2}$) are indicated. (**f**) Predicted glycan holes on ConM SOSIP.v7 and ConM SOSIP.v9.0 trimers. (**g**) Overview of trimer production yield (mg/l), morphology as determined by NS-EM, thermostability and glycan composition.

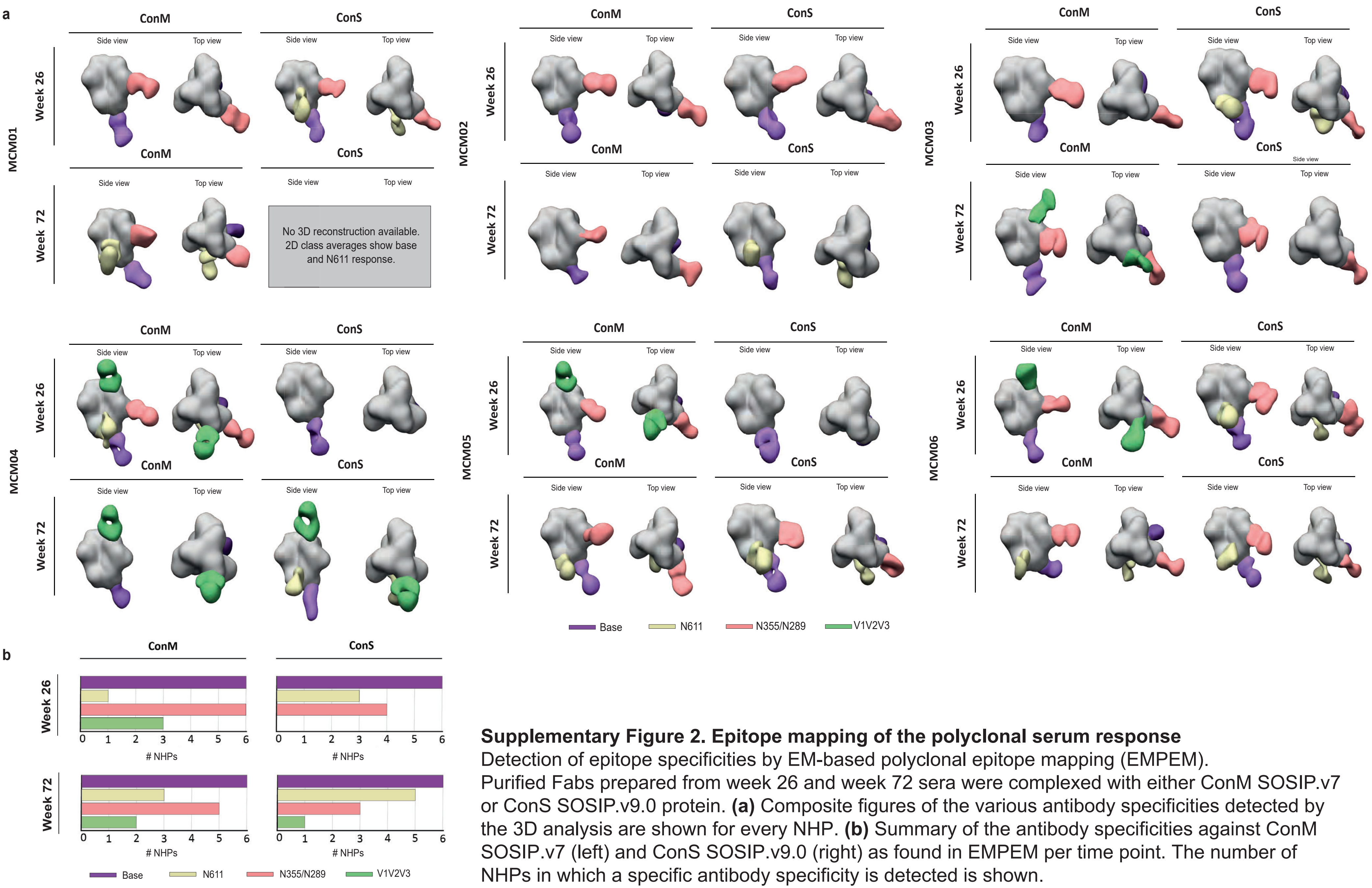

Supplementary Figure 2. Epitope mapping of the polyclonal serum response
Detection of epitope specificities by EM-based polyclonal epitope mapping (EMPEM). Purified Fabs prepared from week 26 and week 72 sera were complexed with either ConM SOSIP.v7 or ConS SOSIP.v9.0 protein. **(a)** Composite figures of the various antibody specificities detected by the 3D analysis are shown for every NHP. **(b)** Summary of the antibody specificities against ConM SOSIP.v7 (left) and ConS SOSIP.v9.0 (right) as found in EMPEM per time point. The number of NHPs in which a specific antibody specificity is detected is shown.

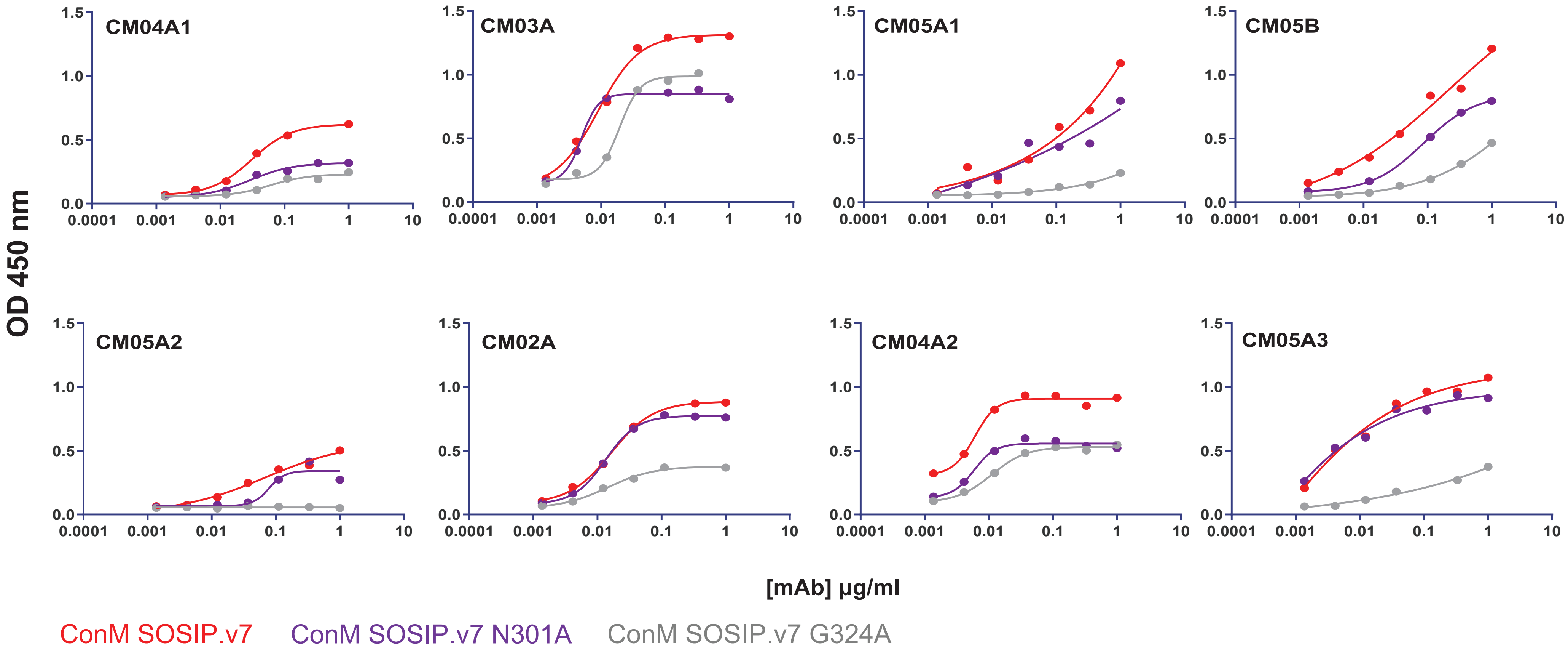

Supplementary Figure 3. Reduced binding of V1V2 region targeting mAbs against G324A and N301 mutants
Eight V1V2 region targeting mAbs were tested in Lectin-capture ELISA for binding to ConM SOSIP.v7 (red), ConM SOSIP.v7 N301A (magenta) and ConM SOSIP.v7 G324A (grey) proteins.

	V1V2 targeting NAbs	
bNAbs	CM02A biotin	CM05A1 biotin
**V1V2 bNAbs**		
PG16	97	88
VRC26.25	104	83
**N332 bNAbs**		
PGT121	51	53
PGT125	55	62
PGT128	75	79

Residual binding (%)

- <25
- 25-49
- 50-74
- >74

Supplementary Figure 4. Competition between isolated V1V2 NAbs and known V1V2 and N332 bNAbs.

Percentage of residual binding of V1V2 and N332 targeting bNAbs to ConM SOSIP.v7 trimers in presence of a competing biotinylated V1V2 targeting NAb (CM05A1, CM02A). Competition was defined as < 60% residual binding with either of the two competitor NAbs.

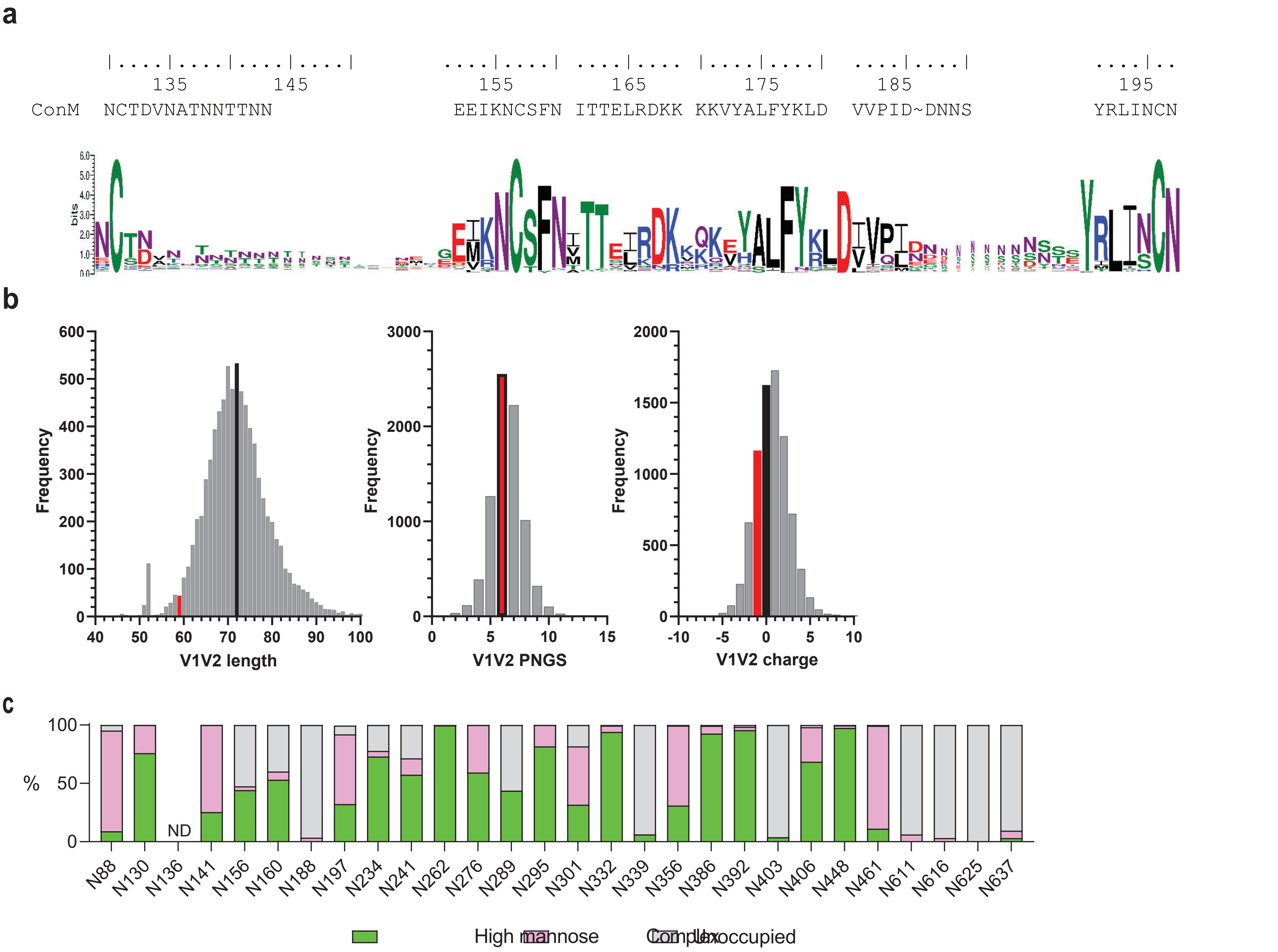

Supplementary Figure 5. ConM SOSIP.v7 V1V2 characteristics and variation

(**a**) The V1V2 sequence of ConM (top) and the weblogo of the V1V2 region (130-197) of all env sequences in the 2020 webalignment, made using http://weblogo.threeplusone.com/create.cgi. (**b**) The length, the number of PNGS and charge of the V1V2 region (130-197) of all env sequences in the 2020 webalignment from the Los Alamos HIV Databases (http://www.hiv.lanl.gov/), determined using the AnalyzeAlign tool of the Los Alamos HIV Databases. In red the length, number of PNGS and charge of the ConM env and in black the median of all env sequences in the 2020 webalignment. (**c**) Site-specific N-linked glycosylation of ConM SOSIP.v7 trimer. The graphs summarize quantitative mass spectrometric analysis of the glycan population present at individual N-linked glycosylation sites. Colors indicate High Mannose (green), Complex (pink) or Unoccupied (grey) sites.

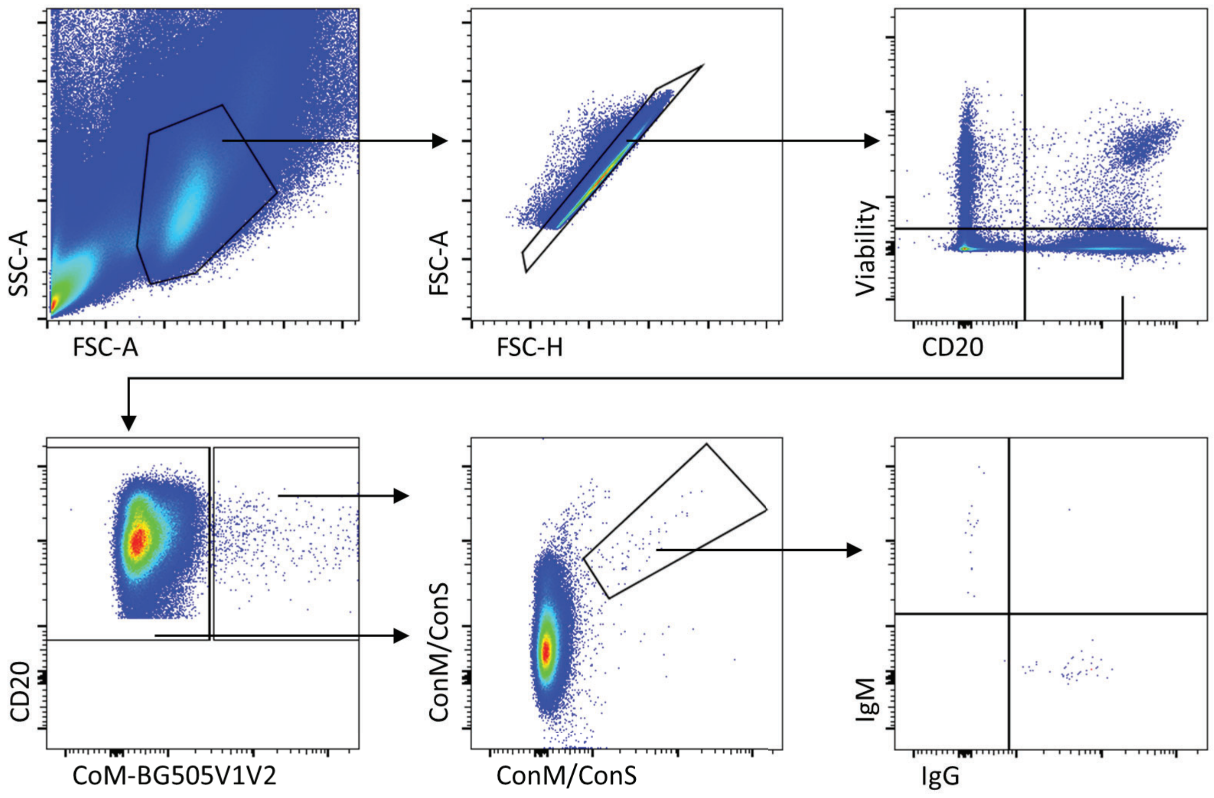

Supplementary Figure 6. Flow Cytometry gating strategy

~10 million PBMCs were stained with all conjugated ConM SOSIP.v7, ConSOSL.UFO.664, and ConM-BG505V1V2 Env proteins, Via eF780 (Invitrogen), IgM BV605 (BioLegend), IgG PE-cy7 (BD Horizon), CD20 PE-CF594 (BD Horizon), and CD27 PE (BD Horizon). Lymphocytes were selected, followed by singlets selection. Viable B cells were selected from the singlets, followed by gating on ConM-BG505V1V2 switch protein negative and positive from which both the ConM/ConS positive cells were selected. These were observed for IgM or IgG expression. ConM/ConS positive cells were sorted from the $CD20^{+}$,ConM-BG505V1V2^{-} gate. The lower 3 panels are shown in **Fig. 2** to vizualize the sorted cells.

Antibody ID	V-gene	CDR3 heavy chain length	CDR AA junction heavy chain	Heavy V-region 160X/Gibson (IMGT)	CDR3 light chain length	CDR AA junction light chain	Light V-region 160X/Gibson (IMGT)
**MCM01**							
CM01A	IGHV5-157*01_S8626	19	CAKSALYYNYWSPYGQGLYFW	Macmul IGHV5-2*01 F	8	CMQVRQYPTF	Macmul IGKV2S17*01 F
CM01B	IGHV4-NL_1*02_S2201	14	CARLPSIMGTTLFDHW	Macmul IGHV4-2*01 F	11	CAAWDDSLSAWLF	Macmul IGLV1-15*01 F
CM01C	IGHV4-NL_41*01_S9253	14	CAREFCSSTYCSQPYW	Macmul IGHV4-2*01 F	9	CQSYDISLSAF	Macmul IGLV1S2*01 F
CM01D	IGHV3-NL_1*01_S6560	18	CTRADRFWNSYSWRIALDSW	Macmul IGHV3-7*01 F	10	CLLYFSGVRVSF	Macmul IGLV7-2*01 F
**MCM02**							
CM02A	IGHV3-187*01_S6641	14	CTRDLWLGYDNSAGYW	Macmul IGHV3-18*01 F	9	CMQETYYPHSF	Macmul IGKV2S17*01 F
CM02B	IGHV3-76*01_S9591	18	CARGLWSGYTPQHTTPLFYW	Macmul IGHV3-14*01 F	9	CQQYDNVPFTF	Macmul IGKV1-20*01 F
CM02C	IGHV3-NL_17*01_S0120	14	CTRGNPMYYSPYFAYW	Macmul IGHV3-13*01 F	9	CFLDESDTLLF	Macmul IGLV7-2*01 F
CM02D	IGHV3-NL_1*01_S6560	15	CTRDSTSGWTNNFFHSW	Macmul IGHV3-7*01 F	9	CQQYNSVPRTF	Macmul IGKV1S6*01 F
CM02E	IGHV4-NL_22*01_S2393	17	CARERWAPNFGLDSW	Macmul IGHV4-2*01 F	9	CQQYNTEPFTF	Macmul IGKV1-22*01 F
**MCM03**							
CM03A	IGHV1-138*01_S2054	21	CARVRGPRYYNFWSGSEISFDYW	Macmul IGHV3-22*01 F	11	CSSYPGSDTDVLF	Macmul IGLV2S2*01 F
CM03B	IGHV3-73*02_S4889		CTRDWGVVQRGLGFCSGPDCYGWFDVW	Macmul IGHV3-7*01 F	13	CATNYGTGSTWHYIF	Macmul IGLV3-19*01 F
CM03C	Could not be aligned	12	CARRRLTGTYIIWW	Macmul IGHV4-2*01 F	9	CQQYHDLLYTF	Macmul IGKV3S1*01 F
CM03D	IGHV3-76*01_S9591	19	CARDRDNFYGSGFYVGHYDSW	Macmul IGHV3-9*01 F	8	CGQGTHLPTF	Macmul IGKV2S4*01 F
**MCM04**							
CM04A1	IGHV3-122*01_S8388	18	CTREGGMGGVIRPYYGLDSW	Macmul IGHV3-9*01 F	9	CQHYYIMPLTF	Macmul IGKV1S22*01 F
CM04B	IGHV3-36*01_S6740	9	YCARDRAMEFW	Macmul IGHV3-9*01 F	9	CLQYNSAPFTF	Macmul IGKV1-9*01 F
CM04C	IGHV4-NL_37*01_S0997	17	CARQFRIAPGLRSWAFDFW	Macmul IGHV4-2*01 F	10	CCSYSGGYSGLF	Macmul IGLV2-8*01 F
CM04D	IGHV5-NL_1*02_S5699	14	CATPPDDYGSGYFYYW	Macmul IGHV5-2*01 F	12	CQSYDSTLTAHVLF	Macmul IGLV1-7*01 F
CM04E	IGHV4-NL_1*01_S9508	14	CARRVTSVTTVWFDVW	Macmul IGHV4-2*01 F	9	CQSYDSSGVLF	Macmul IGLV6-5*01 F
CM04F	IGHV4-NL_40*01_S9020	8	CARFPGRHVW	Macmul IGHV4-2*01 F	9	CQQYNTAPFTF	Macmul IGKV1S6*01 F
CM04G	IGHV3-NL_17*01_S0238	13	CTTDSPYYPRTLYSW	Macmul IGHV3-13*01 F	11	CAAWDDSLSSVLF	Macmul IGLV1S4*01 F
CM04A2	IGHV3-122*01_S8388	18	CAREGALGGVVRPYYGLDSW	Macmul IGHV3-9*01 F	9	CQHYYIMPLTF	Macmul IGKV1-20*01 F
CM04A3	IGHV3-122*01_S8388	18	CTREGGMGGVIRPYHGLDSW	Macmul IGHV3-9*01 F	9	CQQYFSVPLSF	Macmul IGKV1-20*01 F
CM04H	IGHV3-141*01_S8690	19	CAKDLSPAYYWSSSAKFFDVR	Macmul IGHV3-7*01 F	9	CQHGYGVPYSF	Macmul IGKV1-20*01 F
CM04J	IGHV1-138*01_S0032	14	CARADYGNFVGALDFW	Macmul IGHV1-1*01 F	9	CLQHNAYPLTF	Macmul IGKV1S7*01 F
**MCM05**							
CM05A1	IGHV4-NL_16*01_S2138	15	CARGGGGTYCHNSLDVW	Macmul IGHV4-2*01 F	11	CSAWDTSQKTYIF	Macmul IGLV1S7*01 F
CM05B	IGHV4-117*01_S6852	14	CARVPQRLVHLYFDFW	Macmul IGHV4-2*01 F	9	CQHYYGSPWTF	Macmul IGKV1S17*01 F
CM05A2	IGHV4-NL_16*01_S2138	15	CARGGGGTYCHNSLDVW	Macmul IGHV4-2*01 F	11	CSAWDSSLSAYIF	Macmul IGLV1S7*01 F
CM05C	IGHV1-138*01_S2054	19	CARTRGHNFSTGYSLSASDYW	Macmul IGHV1-1*01 F	9	CQQYSRSPFTF	Macmul IGKV3-1*01 F
CM05D	IGHV3-187*01_S6641	21	CTRVRSYYSGSGLYTGSWRFDVW	Macmul IGHV3-18*01 F	10	CCSYRSGSIWVF	Macmul IGLV2-3*01 F
CM05E	IGHV4-NL_18*01_S3907	20	CARNDLAHYYGSDYYGNGFDSW	Macmul IGHV4-2*01 F	9	CQHSYGSPYTF	Macmul IGKV1S9*01 F
CM05A3	IGHV4-NL_16*01_S2138	15	CARGGGGTFCHNSLDVW	Macmul IGHV4-2*01 F	11	CSAWDISLHSYIF	Macmul IGLV1S7*01 F
CM05F	IGHV1-138*01_S2734	17	CARGRPCGAATGCYFLDFW	Macmul IGHV1-1*01 F	10	CLSSDSGLRGLF	Macmul IGLV1-14*01 F
CM05G	IGHV3-177*01_S8409	17	CVKGFILVLPGIARGLDSW	Macmul IGHV3-14*01 F	9	CAIWHNNAVLF	Macmul IGLV5-13*01 F
CM05H	IGHV3-172*01_S8488	11	CARDDWGYGLDSW	Macmul IGHV3-12*01 F	9	CQQGYNTPWTF	Macmul IGKV1S26*01 F
CM05J	IGHV3-122*01_S8388	16	CGKNRCTPNGCFGIFEAW	Macmul IGHV3-9*01 F	9	CQTWDTGIGLF	Macmul IGLV4S2*01 F
CM05K	IGHV1-84*01_S2078	16	CARDGGDDFGFVSSLDVW	Macmul IGHV1-1*01 F	9	CFQYATSPFTF	Macmul IGKV1-21*01 F
CM05L	IGHV1-138*01_S6073	15	CARVGASSGWYWALDSW	Macmul IGHV1-1*01 F	9	CQQRHSYPHNF	Macmul IGKV1-11*01 F
**MCM06**							
CM06A	IGHV3-76*01_S9591	12	CARDRSHYGRFDYW	Macmul IGHV3-14*01 F	10	CSSYAGSNTWVF	Macmul IGLV2S3*01 F
CM06B	IGHV4-NL_5*01_S6960	15	CARLRNVPSATYGLDSW	Macmul IGHV4-2*01 F	9	CMQSLEYPFTF	Macmul IGKV2S17*01 F
CM06C	IGHV1-138*01_S6073	17	CARVSYGKSRALSYGLDFW	Macmul IGHV1-1*01 F	9	CQETGNLSRTF	Macmul IGKV3-5*01 F

Supplementary Table 1. IgDiscover analysis of isolated mAbs

Heavy chain variable (V) gene assignment for each of the isolated mAbs. Heavy and light chain CDR3 amino acid lengths and sequences. Two antibody lineages displaying V-gene similarities are depicted in pink (lineage CM04A) and blue (lineage CM05A).

	Antibody ID	EC_{50} (µg/ml) ConM SOSIP.v7	EC_{50} (µg/ml) ConSOSL.UFO.664
Time point one (week 25)	CM01A	< 0.1	> 100
	CM01B	≥ 0.1 - ≤ 1	≥ 0.1 - ≤ 1
	CM01C	> 100	≥ 0.1 - ≤ 1
	CM03A	< 0.1	< 0.1
	CM03B	< 0.1	< 0.1
	CM04A1	≥ 1 - ≤ 3	< 0.1
	CM04B	< 0.1	≥ 0.1 - ≤ 1
	CM04C	< 0.1	< 0.1
	CM04D	≥ 0.1 - ≤ 1	≥ 0.1 - ≤ 1
	CM04E	> 100	> 100
	CM04F	< 0.1	< 0.1
	CM04G	≥ 1 - ≤ 3	≥ 0.1 - ≤ 1
	CM05A1	< 0.1	≥ 0.1 - ≤ 1
	CM05B	< 0.1	< 0.1
	CM05A2	< 0.1	≥ 0.1 - ≤ 1
	CM05C	< 0.1	≥ 0.1 - ≤ 1
	CM05D	< 0.1	< 0.1
	CM05E	< 0.1	< 0.1

Note: EC_{50} values could not be properly calculated for CM01C and CM04E due to the shape of the binding curve.

	Antibody ID	EC_{50} (µg/ml) ConM SOSIP.v7	EC_{50} (µg/ml) ConSOSL.UFO.664
Time point two (week 71)	CM01D	< 0.1	> 100
	CM02A	< 0.1	≥ 0.1 - ≤ 1
	CM02B	< 0.1	< 0.1
	CM02C	< 0.1	< 0.1
	CM02D	< 0.1	< 0.1
	CM02E	< 0.1	< 0.1
	CM03C	≥ 1 - ≤ 3	≥ 0.1 - ≤ 1
	CM03D	< 0.1	< 0.1
	CM04A2	≥ 0.1 - ≤ 1	< 0.1
	CM04A3	≥ 0.1 - ≤ 1	< 0.1
	CM04H	≥ 0.1 - ≤ 1	< 0.1
	CM04J	< 0.1	≥ 0.1 - ≤ 1
	CM05A3	< 0.1	≥ 0.1 - ≤ 1
	CM05F	< 0.1	< 0.1
	CM05G	≥ 1 - ≤ 3	≥ 0.1 - ≤ 1
	CM05H	≥ 0.1 - ≤ 1	≥ 0.1 - ≤ 1
	CM05J	< 0.1	< 0.1
	CM05K	≥ 1 - ≤ 3	< 0.1
	CM05L	< 0.1	< 0.1
	CM06A	< 0.1	< 0.1
	CM06B	< 0.1	< 0.1
	CM06C	< 0.1	< 0.1

EC_{50} (µg/ml)
< 0.1
≥ 0.1- ≤ 1
≥ 1 - ≤ 3
> 100

Supplementary Table 2. Binding of isolated mAbs to ConM SOSIP.v7 and ConSOSL.UFO.664

Binding of isolated mAbs to ConM SOSIP.v7 and ConSOSL.UFO.664 protein was measured in ELISA.
EC50 values indicate antibody concentrations (µg/ml) at which 50% of the mAb was bound to the respective Env proteins.

	IC$_{50}$ (µg/ml)									
Antibody ID	SVA-MLV	Ce1176.A3	TRO.11	2570-2.43	BJOX002000.03.2	X1632-S2-B10	246-F3_C10_2	CH119.10	Ce703010217_B6	CNE55
CM01A	>50	>50	>50	>50	>50	>50	>50	>50	>50	>50
CM01B	>50	>50	>50	>50	>50	>50	>50	>50	>50	>50
CM02A	>50	>50	>50	>50	>50	>50	>50	>50	>50	>50
CM03B	>50	>50	>50	>50	>50	>50	>50	>50	>50	>50
CM05A1	>50	>50	>50	>50	>50	>50	>50	>50	>50	>50

IC$_{50}$ (µg/ml)
<0.33
≥ 0.33 - ≤ 3
>3 - ≤ 20
>20
No neutralization

Supplementary Table 3. Heterologous neutralization ability of a selection of NAbs

The neutralization activity of three V1V2 targeting NAbs and two non-V1V2 targeting NAbs was tested against a virus panel representing global HIV-1 diversity. The half maximal inhibitory concentration (IC50) values by which the NAbs neutralize the different virus strains are shown.

NS-EM				
**Antibody ID**	**Time point**	**Immunogen**	**Epitope**	**EMDB code**
CM01B	Week 25	ConM SOSIP	V1V2V3	EMD-14888
CM05A1	Week 25	ConM SOSIP	V1V2V3	EMD-14889
CM02A	Week 71	ConM SOSIP	V1V2V3	EMD-14890
CM04F	Week 25	ConM SOSIP	N611	EMD-14891
CM03B	Week 25	ConM SOSIP	Base	EMD-14892
CM05E	Week 25	ConM SOSIP	Base	EMD-14893
CM05L	Week 71	ConM SOSIP	N611	EMD-14894

EMPEM				
**NHP ID**	**Time point**	**Immunogen**	**Epitope**	**EMDB code**
MCM02	Week 26	ConM SOSIP	N355/N289	EMD-14895
MCM04	Week 26	ConM SOSIP	Base	EMD-14896
MCM04	Week 26	ConM SOSIP	N611	EMD-14897
MCM05	Week 26	ConM SOSIP	V1V2V3	EMD-14898
MCM06	Week 26	ConS SOSIP	Base and N611	EMD-14899
MCM02	Week 26	ConS SOSIP	N355/N289	EMD-14900
MCM05	Week 72	ConM SOSIP	Base	EMD-14901
MCM06	Week 72	ConM SOSIP	N355/N289	EMD-14902
MCM06	Week 72	ConM SOSIP	N611	EMD-14903
MCM04	Week 72	ConM SOSIP	V1V2V3	EMD-14904
MCM03	Week 72	ConS SOSIP	Base	EMD-14905
MCM04	Week 72	ConS SOSIP	N611	EMD-14906
MCM04	Week 72	ConS SOSIP	V1V2V3	EMD-14907
MCM06	Week 72	ConS SOSIP	N355/N289	EMD-14908

Supplementary Table 4. EMDB accession codes
Negative-stain EM 3D reconstructions have been deposited to the Electron Microscopy Data Bank (EMDB). EMDB accession codes are provided for individual isolated mAbs (EMD-14888 – 14894), corresponding to figures 4D, 4E, 5B and 5C, and reconstructions per animal, per time point (EMD-14895 – 14908) as shown in figure 1D.
